# Comparative Analysis of the Prevalence of Bovine Viral Diarrhea Virus in Cattle Populations Based on Detection Methods: A Systematic Review and Meta-Analysis

**DOI:** 10.3390/pathogens12081067

**Published:** 2023-08-21

**Authors:** Gebremeskel Mamu Werid, Farhid Hemmatzadeh, Darren Miller, Michael P. Reichel, Yohannes E. Messele, Kiro Petrovski

**Affiliations:** 1 Davies Livestock Research Centre, School of Animal & Veterinary Sciences, University of Adelaide, Roseworthy Campus, Roseworthy, SA 5371, Australia; gebremeskelmamu.werid@adelaide.edu.au (G.M.W.); darren.miller@adelaide.edu.au (D.M.); yohannes.messele@adelaide.edu.au (Y.E.M.); 2 Australian Centre for Antimicrobial Resistance Ecology, School of Animal & Veterinary Sciences, University of Adelaide, Roseworthy Campus, Roseworthy, SA 5371, Australia; farhid.hemmatzadeh@adelaide.edu.au; 3 Department of Population Medicine and Diagnostic Sciences, Cornell University College of Veterinary Medicine, Ithaca, NY 14853, USA; mreichel@hotmail.com

**Keywords:** bovine viral diarrhoea virus, antibody, antigen, nucleic acid and persistent infection

## Abstract

Infectious diseases of cattle, including bovine viral diarrhea (BVD), pose a significant health threat to the global livestock industry. This study aimed to investigate the prevalence and risk factors associated with bovine viral diarrhea virus (BVDV) infections in cattle populations through a systematic review and meta-analysis. PubMed, Web of Science, and Scopus were systematically searched for relevant articles reporting the prevalence of and associated risk factors in studies published between 1 January 2000 and 3 February 2023. From a total of 5111 studies screened, 318 studies were included in the final analysis. BVDV prevalence in cattle populations was estimated using various detection methods. The analysis detected heterogeneity in prevalence, attributed to detection techniques and associated risk factors. Antibody detection methods exhibited a higher prevalence of 0.43, reflecting the cumulative effect of detecting both active and past infections. Antigen detection methods showed a prevalence of 0.05, which was lower than antibody methods. A prevalence of 0.08 was observed using nucleic acid detection methods. The health status of the examined cattle significantly influenced the prevalence of BVDV. Cattle with bovine respiratory disease complex (BRDC) exhibited higher antibody (prevalence of 0.67) and antigen (prevalence 0.23) levels compared to cattle with reproductive problems (prevalence 0.13) or diarrhea (prevalence 0.01). Nucleic acid detection methods demonstrated consistent rates across different health conditions. Age of cattle influenced prevalence, with higher rates in adults compared to calves. Risk factors related to breeding and reproduction, such as natural or extensive breeding and a history of abortion, were associated with increased prevalence. Coinfections with pathogens like bovine herpesvirus-1 or *Neospora caninum* were linked to higher BVDV prevalence. Management practices, such as commingling, introducing new cattle, and direct contact with neighboring farms, also influenced prevalence. Herd attributes, including larger herd size, and the presence of persistently infected cattle, were associated with higher prevalence. These findings indicated the importance of detection methods and risk factors in BVDV epidemiological studies.

## 1. Introduction

Bovine viral diarrhea virus (BVDV) is a significant global cattle pathogen that causes a wide range of health issues, including gastrointestinal and respiratory disorders, reproductive complications, immunosuppression, and, in severe cases, fatal mucosal disease [1,2,3]. The ability of BVDV to cause persistent infection (PI), in which calves infected in utero maintain a lifelong viremia and act as constant viral shedders, is problematic because it contributes to a cycle of transmission within the herd [4,5].

Given the significant economic impact of BVDV [6], accurate estimation of its prevalence is a critical component in devising and implementing effective control and eradication strategies. Diagnostics and vaccines are an integral part of BVDV control programs. A reliable diagnostic method not only detects infected animals early, enabling timely intervention and preventing further transmission, but also monitors vaccination efficacy, differentiates between infected and vaccinated animals, tailors control measures to specific situations, and guides surveillance and eradication efforts [7,8,9]. Hence, selecting diagnostic methods that best serve the purpose and vaccination work together to maximize BVDV control strategies, reduce disease burden, and improve cattle health. However, due to the wide range of available detection methods, intricate risk factors of the virus and challenges associated with differentiating between transiently infected (TI) and PI cattle, the task of estimating prevalence is complicated [10,11,12].

Diagnostic methods, such as serological and nucleic acid detection tests, are among the most commonly used methods to detect BVDV [13,14]. Antibody detection methods, while the sensitivity and specificity vary depending on the type of assay [15], are capable of detecting both current and previous BVDV infections, while antigen and nucleic acid detection assays are typically more indicative of current infections [13,14]. Each diagnostic approach possesses its own advantages and disadvantages, and these differences can considerably affect the observed prevalence in cattle populations [16,17]. Therefore, to ensure accurate representation and understanding of BVDV epidemiology, careful consideration must be given to the diagnostic method selection.

The estimated prevalence range of BVDV varies across different geographic regions and cattle populations [18,19]. It is unclear how much of this variation is a product of differences in detection methods, risk factors, or related covariates. Additionally, there is a limited analysis that combines the data from these studies and provides a comprehensive picture of the prevalence of BVDV using various detection techniques.

To estimate the prevalence of BVDV in cattle populations globally, using antibody, antigen, and nucleic acid detection methods, we carried out a systematic review and meta-analysis of studies published between 2000 and 2023. To explore the BVDV landscape, in addition to animal- and herd-level BVDV prevalence, this analysis closely examined the prevalence of PI animals and the rate of BVDV detection in clinical samples. This study addressed various detection methods and associated risk factors of BVDV prevalence. The findings from this study may provide insights with potential use in the development of reliable benchmarks for effective intervention programs.

## 2. Materials and Methods

### 2.1. Literature Search Strategy

The Preferred Reporting Items for Systematic Reviews and Meta-Analyses (PRISMA) guidelines were followed in developing and implementing the research questions, search, and screening protocols, and in reporting the results [20]. A uniform search strategy was developed and tested across three different databases: Web of Science, PubMed, and Scopus, aiming to identify the relevant literature. The general search string encompassed the terms: “bovine viral diarrhea virus”, “bovine viral diarrhoea virus”, “bovine pestivirus”, and “BVDV”, in conjunction with “cattle”, “bovine”, or “*Bos taurus*”. The search was time-bound to articles published between 1 January 2000 and 3 February 2023.

For the Web of Science, the search topic (TS) was constructed as follows: (((bovine viral diarrhea virus) OR (bovine viral diarrhoea virus) OR (Bovine pestivirus) OR (BVDV)) AND ((cattle) OR (bovine) OR (*Bos taurus*))). For PubMed, the search was executed using the following terms: ((“bovine viral diarrhea virus” (Title/Abstract) OR “bovine viral diarrhoea virus” (Title/Abstract) OR “Bovine pestivirus” (Title/Abstract) OR BVDV(Title/Abstract))) AND (cattle(Title/Abstract) OR bovine(Title/Abstract) OR “*Bos taurus*” (Title/Abstract)). The search on Scopus was carried out with the following key in title, abstract, and keywords (TITLE-ABS-KEY): ((“bovine viral diarrhea virus” OR “bovine viral diarrhoea virus” OR “Bovine pestivirus” OR BVDV) AND (cattle OR bovine OR “*Bos taurus*”)). The retrieved articles were then screened based on the following inclusion and exclusion criteria (Table 1). In addition to using the three databases PubMed, Web of Science, and Scopus, we also implemented a manual search strategy on Google Scholar, utilizing the snowballing method, to broaden our reach and capture any potentially missed studies, thereby ensuring a comprehensive and inclusive research process.

### 2.2. Data Extraction and Quality Assessment

From each of the included articles, a comprehensive set of parameters was systematically extracted, including the author(s) of the study, the year of publication, the geographical origin of the study (country), the type of farming system (beef, dairy, or other types), the design of the study, the type of sample used, and the methodology employed for BVDV detection. The total number of cattle or herds subjected to testing, the total number of cattle or herds found positive for BVDV, the age distribution of the cattle studied, the number of cattle or herds evaluated for persistent BVDV infection, and the number of cattle or herds diagnosed with persistent BVDV infection were also extracted. The quality of studies included was assessed using the Joanna Briggs Institute Qualitative Assessment and Review Instrument tool (JBI-QARI, available at https://jbi.global/critical-appraisal-tools, accessed on 1 May 2023).

### 2.3. Qualitative Data Selection and Analysis

For the analysis of qualitative data, all eligible studies were imported into the NVivo software package [21] for detailed examination and evaluation. These included studies reporting on risk factors and other covariates associated with BVDV detection. Utilizing NVivo, the identified risk factors were systematically selected and coded for categorization. Once this coding process was completed, the refined data were exported for further analysis.

### 2.4. Statistical and Meta-Analysis 

In this study, detection methods were classified into three categories based on the type of analyte they detected. The antibody detection methods utilized were indirect ELISA, competitive ELISA, agar gel immunodiffusion (AGID), and neutralization tests. Antigen detection methods included sandwich ELISA, antigen capture ELISA, immunohistochemistry, direct fluorescent antibody test (DFAT), and direct ELISA. The nucleic acid detection methods comprised reverse transcriptase polymerase chain reaction (RT-PCR), nested RT-PCR, and real-time RT-PCR. Notably, competitive ELISA was categorized either as an antibody or an antigen detection method, contingent on the specific analyte detected in the respective study.

Meta-analysis was carried out utilizing the ‘meta’ and ‘metafor’ packages in R [22]. The principal effect measure was determined as a pooled proportion, which was computed by dividing the number of cattle tested positive for BVDV by the total number of cattle tested, expressed at a 95% confidence interval. To account for the expected heterogeneity among the studies a random effects model in the pooled analysis was applied. This heterogeneity was evaluated using the I^2^ statistic [23] and the Q test [24].

To explore potential sources of heterogeneity, a subgroup analysis based on the type of detection method (antibody, antigen, nucleic acid), the category of animal (beef cattle, dairy cattle, local breeds), and health status (BRDC, reproductive issues, etc.) was conducted. These subgroups were identified based on predefined variables such as intervention type and study design.

The distribution of effect size was also visually inspected using graphic display of heterogeneity (GOSH) plots [25,26]. Given the nonnormal distributions often observed in epidemiological data, the DBSCAN algorithm was selected for outlier detection [26]. Post GOSH analysis, outliers identified using both DBSCAN and influence analysis were systematically removed from the dataset. After outlier exclusion, a sensitivity analysis was carried out to assess the robustness of our findings. The meta-analysis was repeated to ensure the integrity of our results after the removal of outliers.

Prevalence estimates, expressed as a proportion, and their corresponding 95% confidence intervals (CIs) were calculated using the DerSimonian–Laird random effects model. We also assessed the potential presence of publication bias using Peters’ [27] and Egger’s [28] regression tests. A *p*-value of less than 0.05 was considered to indicate statistical significance.

## 3. Results

### 3.1. Study Selection and Characteristics

**Figure 1:**
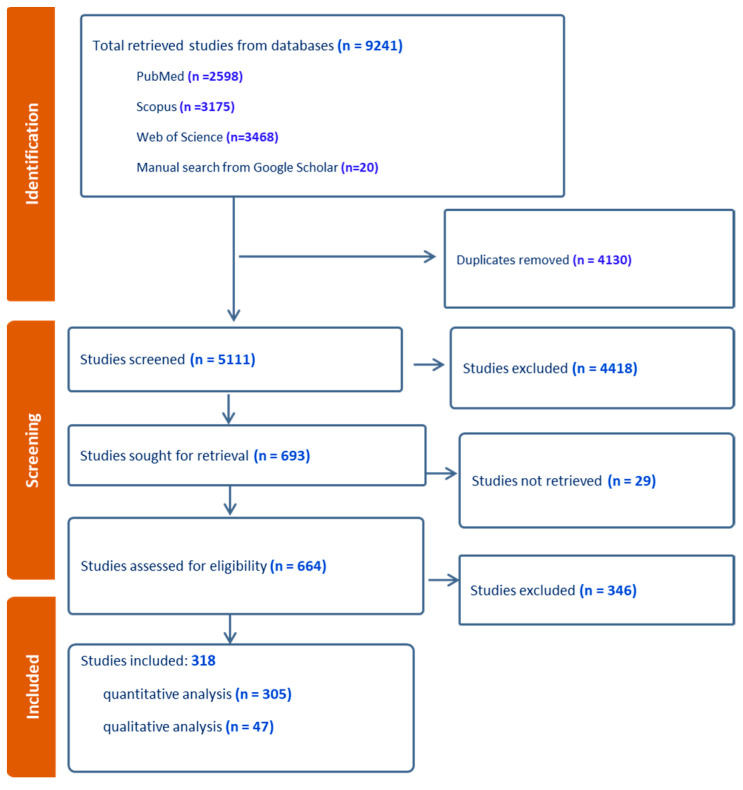
Data collection and screening flow chart.

**Figure 2:**
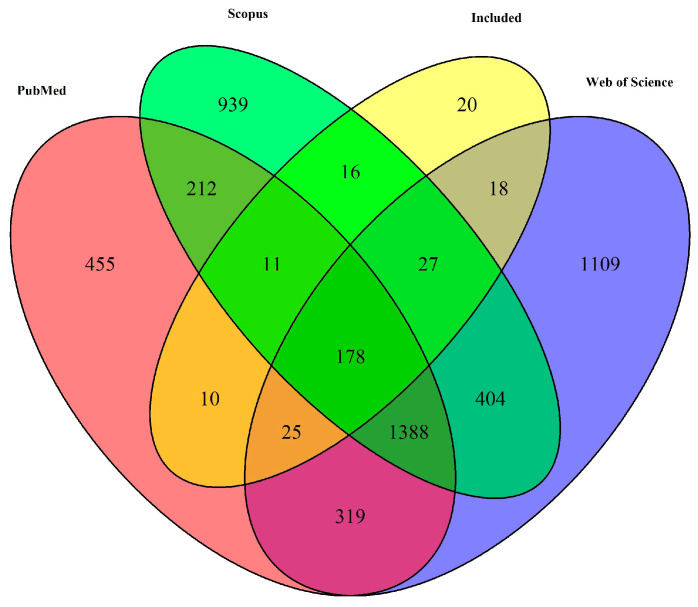
Venn diagram showing overlap of studies from PubMed, Web of Science (WoS), and Scopus databases, including those selected for quantitative analysis (labelled as ‘Included’).

In this study, a total of 9241 studies were initially retrieved, 5111 studies were screened after duplications were removed, and 318 studies were included in the final analysis (Figure 1). Out of the total, 305 studies were chosen for quantitative analysis. An additional 47 studies were selected for qualitative research, among which 34 were also part of the quantitative analysis and the remaining 13 were unique to the qualitative study. An exception to this data extraction process was a group of 20 studies that were manually retrieved from Google Scholar, with the remainder being identified from various databases using specific search terms (Figure 2). The quantitative analysis included studies from 62 countries.

A subgroup of the quantitative analysis comprising 243 studies, henceforth referred to as “randomly selected samples”, was utilized to estimate the pooled prevalence of BVDV. Based on the randomly chosen samples from a subset of cross-sectional and longitudinal studies, a range of BVDV prevalence was determined. The prevalence of BVDV varied depending on the detection method–an antibody, antigen, or nucleic acid. Further statistical analyses revealed a significant influence of the detection method on the observed BVDV prevalence (Table 2). Based on data from 243 studies, and as determined by antibody, antigen, and nucleic acid detection methods, BVDV was found to be distributed globally (Supplementary Figure S1).

In contrast, from the 305 studies, another set of 80 studies that detected BVDV from clinical samples was included for quantitative analysis (Supplementary Materials). These samples were sourced from cattle suspected of having BVDV or those with a history of respiratory, reproductive, and other health issues, particularly those linked with BVDV, such as diarrhea. For analytical purposes, this group was categorized as “clinical samples”. Depending on the type of detection method, differences (*p* < 0.001) were observed in the prevalence of BVDV.

### 3.2. Prevalence of Bovine Viral Diarrhea Using Antibody Detection Methods

For animal-level BVDV prevalence estimation, using antibody detection methods, a total of 144 studies were included. To check the reliability of the pooled BVDV prevalence estimates, using antibody detection methods, a sensitivity analysis was carried out. The GOSH plots and DBSCAN were used to identify outliers in the data. Detected outliers were removed (*n* = 22) and the meta-analysis was rerun to compare the post-outlier removal prevalence (Table 3).

The sensitivity analysis indicated a subtle increase in the prevalence and a reduction in heterogeneity following the removal of outliers (Table 3), indicating an improvement in the study’s precision. However, the heterogeneity metrics remained high, suggesting substantial variability among the included studies. Final pooled prevalence estimation was carried out following outlier exclusion. Subgroup analysis was also carried out to explore the difference in prevalence in the type of farms, study design and type of antibody detection used. Prevalence of BVDV in adult cattle, calves, beef cattle, dairy cattle, and local cattle was found at 0.47, 0.23, 0.45, 0.49, and 0.48, respectively (Supplementary Figure S2A,B). No significant difference was detected between the study design, farm type, and type of antibody detection used. However, age-based difference in the prevalence of BVDV was observed between adult cattle and calves (*p* < 0.0001).

Following removal of outliers, a slight increase in the herd-level BVDV prevalence along with a reduction in heterogeneity was observed (Table 3 and Supplementary Figure S2C).

To assess the presence or absence of publication bias in the included studies, both Peters’ and Egger’s regression detected no bias with Peter’s test yielding a nonsignificant t-statistic of 0.93 (*p* = 0.35, df = 142) and a bias estimate of 10.86. Egger’s test confirmed these findings, with a t-statistic of 0.096 (*p* = 0.92, df = 142) (Supplementary Figure S3). These results indicate a lack of notable funnel plot asymmetry, suggesting meta-analysis results in this study were not influenced by publication bias.

### 3.3. Prevalence of Bovine Viral Diarrhea Using Antigen Detection Methods

From the 305 studies, a total of 46 studies, 6 longitudinal and 40 cross-sectional, were included for the animal-level analysis of the pooled prevalence of BVDV using antigen detection methods (Supplementary Figure S4A,C). In the analysis of BVDV prevalence based on antigen detection methods, there was a significant difference between antigen ELISA and immunohistochemistry-based detection methods, farm type, age of cattle, study design, and farm type.

To check the reliability of the estimated BVDV prevalence, a sensitivity analysis was carried out using GOSH and identified potential outliers were removed from the final analysis (Table 3).

After the removal of outliers, a decrease in the prevalence and heterogeneity measures (Tau^2^, Tau, and I^2^) was observed, providing a more accurate estimation of BVDV prevalence (Table 3).

Using antigen detection methods, the animal level prevalence of BVDV in adult cattle, calves, beef cattle, dairy cattle, and local cattle breeds was 0.06, 0.06, 0.10, 0.06, and 0.0412, respectively (Supplementary Figure S4A,B). Similarly, eight studies based on antigen ELISA reported a herd level of BVDV prevalence and a pooled estimate of 0.33 (95% CI: 0.08; 0.65) was observed (Supplementary Figure S4D).

The presence of publication bias in the studies investigating BVDV using antigen detection methods was evaluated using Egger’s and Peters’ tests. Peters’ test suggested presence of publication bias (t-statistic: 2.58, *p* = 0.01, bias estimate: 61.71). Conversely, Egger’s test indicated lack of publication bias (t-statistic: 0.43, *p* = 0.67, bias estimate: −1.74).

### 3.4. Prevalence of Bovine Viral Diarrhea Virus Using Nucleic Acid Detection Methods

Sensitivity analysis was carried out after removing outliers identified via the GOSH analysis from the meta-analysis on the results of BVDV prevalence using nucleic acid detection methods. This resulted in a change from the initial pool of 50 studies down to 49 (Table 3).

After outlier removal, there was a decrease in prevalence, suggesting that the outliers overestimated the prevalence. Additionally, the breakdown by subgroup, in the final analysis, highlights a differential BVDV prevalence among different cattle populations (Supplementary Figure S5), though no differences were found among these subgroups (*p* > 0.05). When analyzing BVDV prevalence using nucleic acid detection methods, no variation among farm classification type, animal age, study design, or specific detection method was observed. The prevalence of BVDV was highest among local cattle (0.15, 95% CI: 0.00–0.99; *n* = 3 studies) followed by beef cattle (0.10, 95% CI: 0.02–0.24; *n* = 11), dairy cattle (0.07, 95% CI: 0.03–0.14; *n* = 16), and a mixed or unknown category (0.03, 95% CI: 0.01–0.05; *n* = 19) Supplementary Figure S5A–C). The adult cattle population had a prevalence of 0.07 (95% CI: 0.04–0.11; *n* = 37), while the prevalence among calves was slightly lower at 0.06 (95% CI: 0.01–0.15; *n* = 12). Similarly, a herd-level prevalence of BVDV, as determined using nucleic acid detection methods, was 0.28 (95% CI: 0.07–0.54) (Supplementary Figure S5D).

The presence of publication bias in studies investigating BVDV using nucleic acid detection methods was evaluated using Egger’s and Peters’ tests. Peters’ test indicated funnel plot asymmetry (t = −2.20, df = 45, *p* = 0.03), suggesting smaller studies may overestimate effect sizes. Similarly, Egger’s test indicated asymmetry (t = −6.81, df = 48, *p* < 0.00), suggesting larger studies have an effect size of approximately 0.36.

### 3.5. Detection Rates of Persistent Bovine Viral Diarrhea Virus Infection

**Figure 3:**
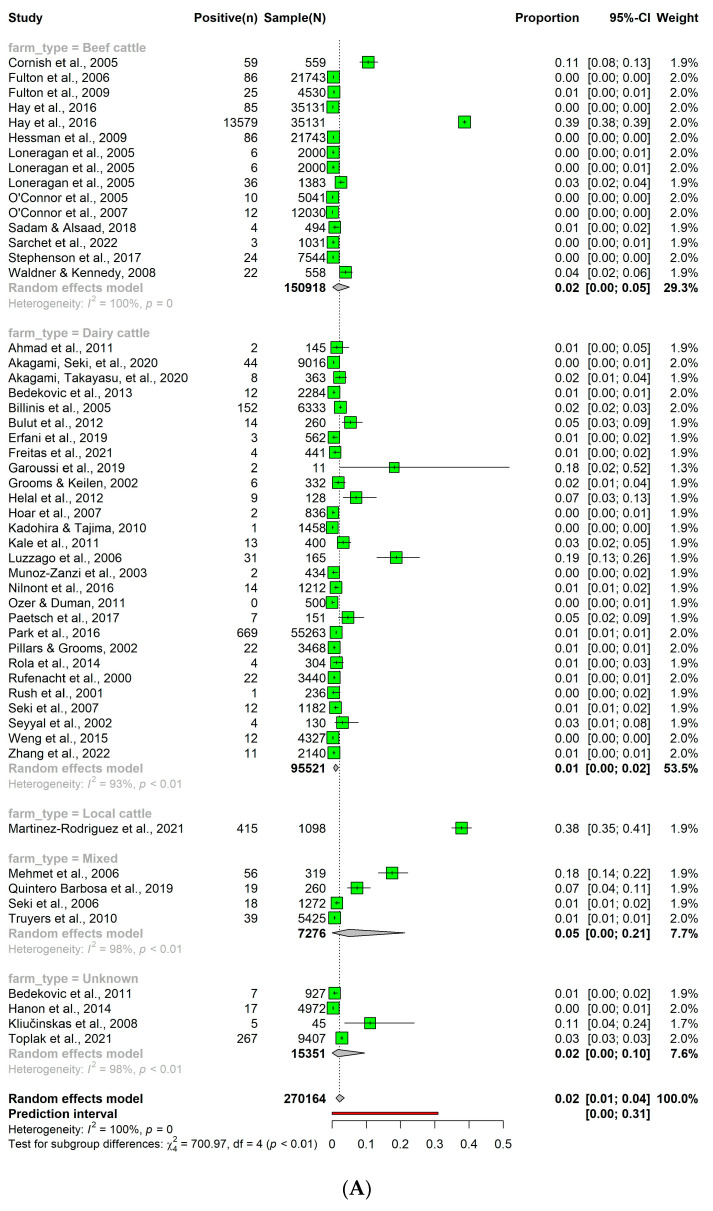

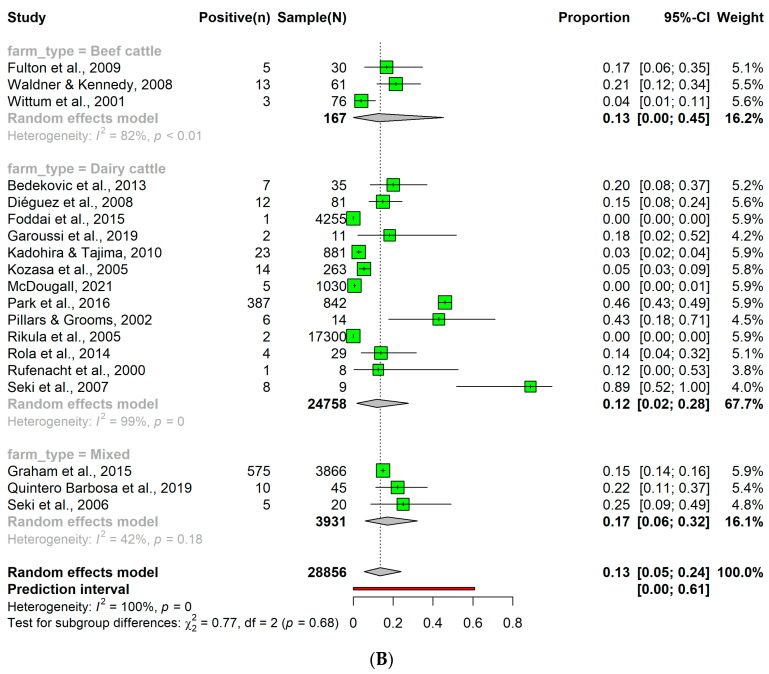
(**A**) Animal level detection rate of persistently infected bovine viral diarrhea virus in cattle. (**B**) Herd level detection rate of persistently infected bovine viral diarrhea virus in cattle.

From the 305 studies, a total of 51 studies focused on cattle level and 19 studies on herd level were included for the analysis of the detection rate of BVDV. A PI cattle prevalence of 0.02 (95% CI: 0.01; 0.04) was observed. Despite numerical variations (Figure 3A) in the prevalence of PI cattle among different subgroups, no statistically significant differences were observed. Similarly, the herd level prevalence of PI in cattle was 0.14 (95% CI: 0.05; 0.24) (Figure 3B).

### 3.6. Detection of Bovine Viral Diarrhea Virus in Clinical Samples

Parallel to the detection of BVDV in randomly selected samples, the prevalence in clinical samples (*n* = 60), referred thereof as the BVDV detection rate, was also dependent on the detection method (*p* < 0.05), indicating the influence of the chosen detection method on the resulting prevalence.

**Figure 4:**
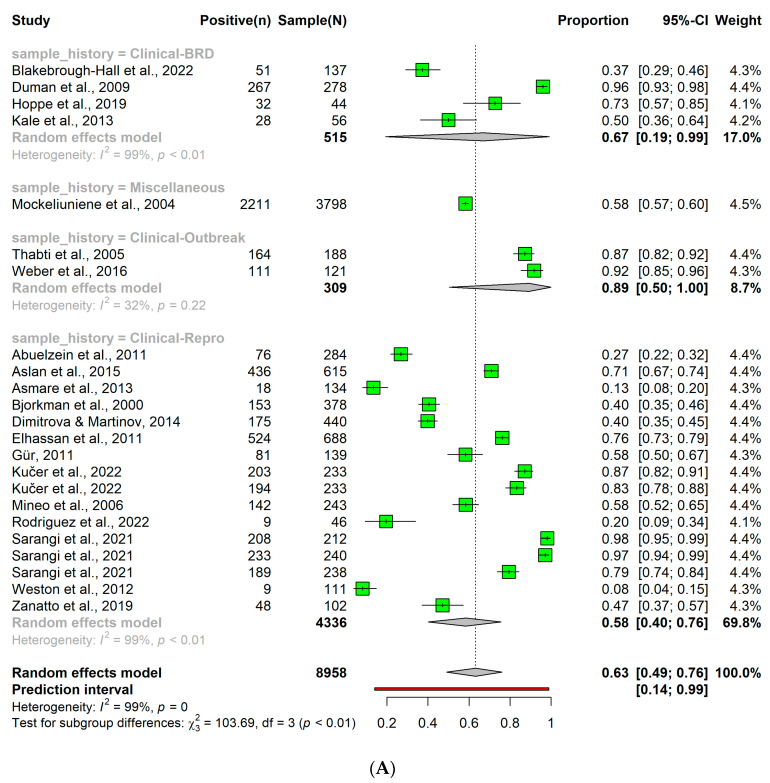

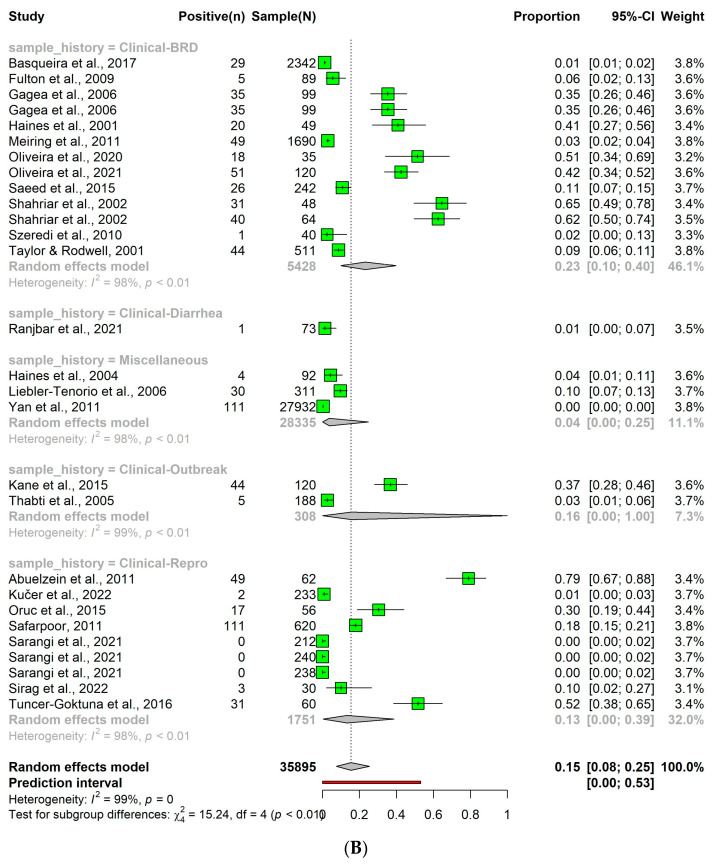

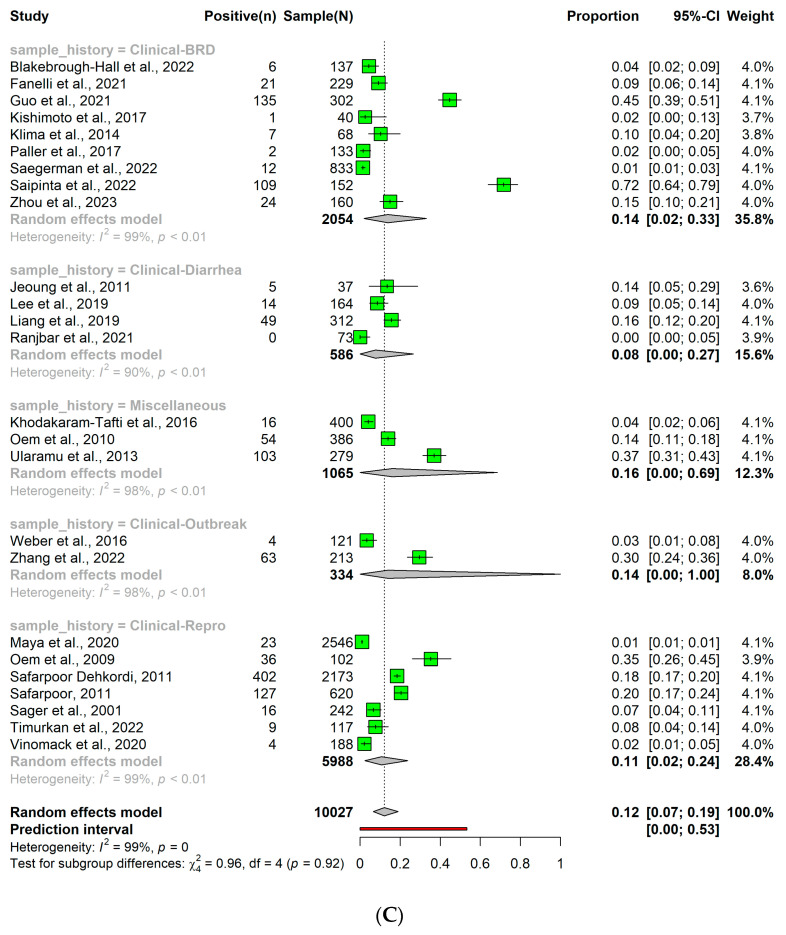
(**A**) Detection rate of bovine viral diarrhea virus in clinical samples using antibody detection method. (**B**) Detection rate of bovine viral diarrhea virus in clinical samples using antigen detection method. (**C**) Detection rate of bovine viral diarrhea virus in clinical samples using nucleic acid detection method.

When the antibody detection method was used, a higher overall BVDV detection rate of 0.63 (95% CI: 0.49; 0.76) was observed (Figure 4A). Subtle variations in the BVDV detection rate were found, contingent on the health conditions of the sample population. Specifically, the BVDV detection rate were 0.67 and 0.58 in cattle exhibiting signs of BRDC or reproductive problems respectively (*p* < 0.05).

Using the antigen detection method in clinical samples, resulted in an overall BVDV detection rate of 0.15 (95% CI: 0.08; 0.25) (Figure 4B). Similar to the antibody detection method, the rate showed subtle differences among the subgroups of the sample population. The BVDV detection rate was 0.23, 0.13, or 0.01 for cattle with signs of BRDC, reproductive problems, or diarrhea, respectively (*p* < 0.05).

In contrast, using the nucleic acid detection method an overall BVDV detection rate of 0.12 (95% CI: 0.07; 0.19), with no variations between health conditions, being 0.14, 0.11, or 0.08 in cattle exhibiting signs of BRDC, reproductive problems, or diarrhea, respectively (*p* > 0.05; Figure 4C). 

Using antibody, antigen, or nucleic acid detection methods, the BVDV detection rate in cattle exhibiting BRDC signs was higher than the other health problems. In clinical samples, antigen and nucleic acid detection methods achieved an overall similar result. Taken together, these findings indicate that both the detection method and the health status of the animal population impacted the observed BVDV detection rate.

### 3.7. Risk Factors and Covariates

The current study observed various risk factors and covariates with potential to influence the prevalence of BVDV. The identified risk factors include age of cattle, coinfection, management practices, and specific demographic factors. Detailed insights into these parameters are presented in Table 4.

## 4. Discussion

We carried out a systematic review and meta-analysis with aims to determine the prevalence of BVDV in cattle populations using a variety of detection methods, such as antibody, antigen, and nucleic acid methods, and investigate associated risk factors. We observed heterogeneity in the prevalence across studies, largely attributable to the variability in the detection methods and risk factors. Our results were consistent with previous reviews and meta-analyses on prevalence of BVDV that reported considerable variations [18,19,76].

The choice of detection method played a crucial role in the observed prevalence of BVDV, with antibody, antigen, and nucleic acid detection methods yielding varying results. Using antibody detection methods, we observed a higher prevalence of BVDV, which can be attributed to the capacity of these methods to detect both current and past infections. This was consistent with prior reports [77,78] suggesting that BVDV infections often elicit robust and long-lasting antibody responses. It is important to note that cattle with PI do not seroconvert in response to the virus, thus remaining undetectable in surveys that depend on antibody-based detection. However, the presence of these PI animals is detectable when using antigen- or nucleic acid-based detection methods [79,80,81]. As a result, antibody-based detection might not offer a comprehensive view of the BVDV epidemiological landscape within cattle populations. The sensitivity analysis supports the reliability of these prevalence estimates, demonstrating that outliers exerted minimal influence on the outcome. In contrast, the prevalence estimates derived from antigen detection methods were markedly lower. This could be explained by the short period of viremia associated with transient BVDV infection [82] and the decline in viremia levels in PI cattle [83], which reduces the detectability of the antigen over time. Even though the prevalence may seem lower with antigen detection methods, these tests are essential in the early identification and isolation of PI cattle. Prevalence determined through nucleic acid detection methods were lower than antibodies but higher than antigen detection methods. These differences may reflect the superior analytical sensitivity and specificity of nucleic acid detection methods [84,85] and the capacity to detect BVDV in the early stages of infection [86]. Nucleic acid methods have generally higher detection rates, compared to antigen or antibody detection methods [79]. Nevertheless, we observed a substantial variation in prevalence estimates among studies utilizing nucleic acid detection methods. Indeed, this may have occurred due to their intrinsic nature [16,87,88]. However, the variation in prevalence of BVDV may have occurred as result of the influence of other factors, including laboratory procedures, sampling strategies, and target populations.

An overall PI cattle detection rate of 0.02 was observed in our study. This was consistent across various cattle populations which is indicative of a stable prevalence of PI cattle. However, herd-level sampling suggested a higher prevalence of 0.14, providing a potentially more precise gauge of BVDV prevalence. Detecting PI cattle, a crucial element in understanding BVDV prevalence, can be achieved through antigen or nucleic acid detection methods. However, confirming PI status and differentiating from acute infection often necessitates retesting in two to four weeks [79,80,81], introducing an additional layer of complexity to the detection process. Both TI and PI animals could become initially positive for the virus via methods such as virus isolation, antigen detection, or nucleic acid detection. However, upon repeating the same test, TI animals, having cleared the virus, will test negative, while PI animals remain positive [77,81]. Since TI animals produce antibodies during post-infection while PI cattle do not, serological tests that detect antibodies could also be used as a differentiating tool. Hence, the use of a strategically combined diagnostic approach can optimize both cost and efficiency.

Using reliable diagnostics for identifying PI cattle and then immediate removal of PI animals from herds is the cornerstone of a successful BVD control program. This step must be completed to protect the herd from future infections [12]. The emphasis switches to vaccination and biosecurity as the main lines of defense against BVDV once a herd has been determined to be PI-free. The economic impact of BVD has spurred many countries, mainly in Europe and the USA, to adopt control and eradication measures [89,90]. Systematic BVDV vaccination supported by active surveillance is recommended in high-risk areas during the first program phases following PI animal removal [11,12].

Detecting BVDV in clinical samples requires careful consideration of both the chosen detection method and the health status of the animal population [77]. In our study, differences in the prevalence of BVDV were observed among the diagnostic methods, and there was a notable influence of the health status of the sampled population. The antibody detection method yielded the highest detection rate of 0.63. However, this prevalence varied depending on the health conditions of the animal. Cattle exhibiting signs or history of BRDC had a prevalence of 0.67, while those presenting with a history of reproductive problems had a slightly lower rate of 0.58. BVDV is known to impact the reproductive performance of cattle [1]. Our findings indicated that cattle with BRDC may exhibit stronger antibody responses, leading to higher prevalence. On the other hand, reproductive problems associated with BVDV might not provoke a strong antibody response, potentially resulting in lower detection rates. This discrepancy may indicate the varying immunological responses elicited by different clinical presentations of BVDV infection.

The efficiency of the antigen detection method varied among different health conditions of the cattle populations involved in this meta-analysis. Similar to the antibody detection methods, cattle exhibiting signs of BRDC had a prevalence of 0.23, while those with diarrhea and reproductive problems were observed with lower prevalence of 0.01 and 0.13, respectively. These findings suggested that antigen detection might be more successful in identifying BVDV in cattle with BRDC, a condition likely associated with active viral replication. However, it appears less effective in cattle with diarrhea or reproductive problems.

In contrast, nucleic acid detection methods provided an overall BVDV prevalence of 0.12. The variations in prevalence among cattle displaying different health conditions were minimal. These findings suggested that nucleic acid detection might be less influenced by the health status of assessed cattle populations. Yet, nucleic acid detection methods demonstrated higher accuracy than antigen-based methods for BVDV detection in fetal specimens [77], highlighting the need to select detection methods based on sample type.

Our findings indicated that both the detection method and the health status of the animal population do influence BVDV prevalence. Across all detection methods, a higher prevalence of BVDV in cattle exhibiting BRDC signs was observed. This could imply that the involvement of BVDV in BRDC is more common than previously thought, at least in the populations we studied. Similarly, the role of BVDV in reproductive problems might have been underestimated. These insights call for further research to expand our understanding of BVDV infection in cattle.

In addition to the detection method, BVDV prevalence was influenced by various risk factors such as the age of cattle, farm management, practices related to breeding and reproduction, and coinfection. The age of cattle was identified as a significant factor influencing seroprevalence of BVDV, being higher in adult cattle compared to calves [29,30,31,32,33]. However, this could be influenced by the detection method used, since older cattle, having had more cumulative exposure to the virus, might show higher seroprevalence, especially when detected via antibody-based assays. Further variations in age-related prevalence are evident, as cows have higher odds of testing seropositive over heifers [36], which might reflect the prolonged exposure or potential re-exposure in breeding females. Certain risk factors related to breeding and reproduction, including natural or extensive breeding [37,39,40,41,42,43], history of abortion [44,45], repeat breeding [46], and the status of being pregnant or lactating [33,47] also correlated with increased prevalence of BVDV. This points to the potential impacts of BVDV on reproductive health, with potentially significant effects on herd productivity, and the importance of regular surveillance. Timing of sample collection is another factor that can affect prevalence of BVDV, with postcolostral serum samples in calves yielding a higher prevalence compared to precolostral samples [34]. This might be due to the potential of maternally derived antibodies in precolostral samples influencing serological test results, particularly in assays that detect BVDV antibodies. 

Coinfections with other pathogens, such as pneumonic *pasteurellosis* [48], bovine herpesvirus-1 [35,39,49,50], and *Neospora caninum* [36,50,51,52], were associated with higher prevalence of BVDV. These findings imply the importance of employing comprehensive diagnostic approaches for considering multiple potential pathogens.

Moreover, the current study highlighted the impact of cattle management practices on prevalence of BVDV. Commingling or introducing new cattle [36,37,53,54,55], purchasing cattle [38,56,57,58,59,60,61,62,63], and direct contact with neighboring farms [41] increased the risk of BVDV detection, likely due to increased potential for pathogen spread. The type and size of herd were also associated with prevalence of BVDV, with certain breeds [42,63,65], larger herds [43,52,58,62,63,66,72,73,74], and the presence of PI cattle in the pen [75] leading to higher prevalence. Again, this may reflect both the complex factors related to infection dynamics and the influence of detection methods. The role of geographical location [39,59,68,70,71] and season [29] on prevalence of BVDV requires consideration when interpreting results from different studies in a meta-analysis.

This study has some limitations that should be considered when interpreting the results. First, the heterogeneity observed among the included studies suggested that factors such as geographic region, detection methods, risk factors, and study design may have influenced the estimates in the prevalence of BVDV. Second, the search strategy was limited to studies published in English, which could have introduced language bias. Third, as studies reporting higher prevalence estimates are more likely to be published, the possibility of publication bias cannot be ruled out.

## 5. Conclusions

Our findings suggest that BVDV remains a widespread and significant pathogen in cattle populations worldwide, with varying prevalence across different regions. This meta-analysis revealed the complex relationships between multiple risk factors and prevalence of BVDV, emphasizing the significant impact of detection methods on reported rates. It also underlines the adaptation of standard and context-specific detection methods in BVDV studies, fostering more precise comparisons and interpretations. Additionally, our analysis proposes the possible advantage of mixed testing strategies to enhance the accuracy of BVDV prevalence estimates. Hence, additional investigations are encouraged to provide a more profound understanding of BVDV epidemiology and transmission dynamics.

## Figures and Tables

**Table 1:** Inclusion and exclusion criteria employed for selection of studies for data gathering for the meta-analysis and systematic review.

Criterion	Description
Inclusion	Study design: Cross-sectional, longitudinal, case reports investigating BVDV in cattle;
	Population: Cattle of any region, breed, age, production system;
	Outcome measures: Prevalence/detection of BVDV, confirmed via laboratory tests;
	Language: English or other languages with translations;
	Sample size: More than 30;
	Publication type: Peer-reviewed articles.
Exclusion	Study design: Case reports with <30 cases, reviews, editorials, commentaries, opinion articles;
	Population: Noncattle species;
	Outcome measures: Studies without BVDV prevalence/detection or lab confirmation;
	Data quality: Studies with significant flaws, unclear reporting, or missing key information;
	Sample size: Less than 30;
	Missing information: On the origin and type of data used for analysis;
	Duplicate data: Studies reporting duplicate/overlapping data.

**Table 2:** Summary of detection rates of bovine viral diarrhea virus based on the detection method.

Detection Method	Number of Pooled Studies	Prevalence	95% CI	Tau^2^	Tau	Q	I^2^
Antigen	46	0.05	[0.02; 0.08]	0.01	0.12	10,585.38	99.6%
Antibody	144	0.43	[0.39; 0.48]	0.05	0.22	44,267.72	99.7%
Nucleic acid	50	0.08	[0.04; 0.11]	0.19	0.43	58,908.83	99.9%
Virus isolation	3	0.22	[0.03; 0.50]	0.01	0.11	23.09	91.3%

**Table 3:** Sensitivity analysis of the prevalence of BVDV using different detection methods.

Detection Method	Outlier Removal Status	Number of Studies	Prevalence	95% CI	Tau^2^	Tau	I^2^
Antibody	Pre-outlier removal	144	0.43	[0.39; 0.48]	0.05	0.22	0.997
	Post-outlier removal	122	0.44	[0.39; 0.49]	0.05	0.21	0.996
Antibody (herd-level)	Pre-outlier removal	40	0.66	[0.54; 0.77]	0.44	0.67	0.999
	Post-outlier removal	36	0.71	[0.62; 0.80]	0.08	0.28	0.98
Antigen	Pre-outlier removal	46	0.05	[0.02; 0.08]	0.01	0.12	0.996
	Post-outlier removal	37	0.03	[0.02; 0.05]	0.01	0.09	0.989
Nucleic acid	Pre-outlier detection	50	0.08	[0.04; 0.11]	0.19	0.43	0.999
	Post-outlier removal	49	0.07	[0.04; 0.10]	0.02	0.13	0.987

**Table 4:** Major risk factors associated with bovine viral diarrhea (BVDV) infection in cattle.

Risk Factors	Description	Reference
Age of cattle	Adult cattle have higher prevalence compared to calves.	[29,30,31,32,33]
	Postcolostral serum samples yielded a higher prevalence compared to precolostral samples.	[34]
	Heifers have a lower seropositive rate compared to calves.	[35]
	Cows have higher odds of testing seropositive over heifers.	[36]
	Weaning age.	[37]
	Number of calves on a farm less than one year old.	[38]
Breeding and reproduction	Natural or extensive breeding.	[37,39,40,41,42,43]
	History of abortion is associated with a higher prevalence.	[44,45]
	Serostatus to BVDV is associated with repeat breeding.	[46]
	Pregnant and lactating cattle.	[33,47]
Coinfection status	Calves with pneumonic *Pasteurellosis*.	[48]
	Concurrent infection with BHV-1.	[35,39,49,50]
	Coinfection with and *Neospora caninum*.	[36,50,51,52]
Cattle mixing	Commingling or cattle introduction.	[36,37,53,54,55]
	Cattle purchase.	[38,56,57,58,59,60,61,62,63]
	Direct contact with neighboring farms.	[41]
	Exposure to persistently infected calves did not affect the prevalence.	[53,64]
Farm management	Farm owner literacy level.	[39]
	Isolation paddocks for ill cattle.	[41]
	Pastureland management.	[38,59,65]
	Not providing colostrum.	[40]
	Cattle management system including housing and hygiene.	[30,36,54,55,66,67]
	Farm size area and animal density.	[37,68]
	Exchange visits between adjacent farm workers.	[60]
Geographical location and season	Season of the year.	[29]
	BVDV infection in a nearby farm.	[62,69]
	Geographic location.	[39,59,68,70,71]
Herd size and type	Breed.	[42,63,65]
	Herd size.	[43,52,58,62,63,66,72,73,74]
	PI cattle in a pen.	[75]
	Distance from positive herds.	[72]

## Data Availability

All datasets used in this study are readily accessible upon request to the authors.

## Supplementary Materials

The following supporting information can be downloaded at: https://www.mdpi.com/article/10.3390/pathogens12081067/s1, Supplementary Figures S1–S5. Supplementary Figure S1. Global distribution of bovine viral diarrhea virus using (Refs.) (A) antibody detection method, (B) antigen detection methods and (C) nucleic acid detection methods. Supplementary Figure S2. (A) Prevalence of bovine viral diarrhea virus using antibody detection methods in subgroups of adult cattle (cattle) and calves. To accommodate the large number of studies included in this analysis, individual studies have been masked on the subplot for optimal page fit. (B) Prevalence of bovine viral diarrhea virus using antibody detection methods in subgroups of Beef, dairy, local and other cattle populations. To accommodate the large number of studies included in this analysis, individual studies have been masked on the subplot for optimal page fit. (C) Herd level prevalence of Bovine viral diarrhea virus using antibody detection methods. Supplementary Figure S3. Publication bias analysis using funnel plots for (A) Antibody detection methods, (B) Antigen detection methods and (C) nucleic acid detection methods. Supplementary Figure S4. (A) Prevalence of Bovine viral diarrhea virus using antigen detection methods in subgroups of adult cattle and calves. (B) Prevalence of bovine viral diarrhea virus using antigen detection methods in various farm-type subgroups. (C) Prevalence of bovine viral diarrhea virus detected using antigen ELISA and immunohistochemistry (IHC) methods. (D) Herd level prevalence of bovine viral diarrhea virus using antigen detection methods. Supplementary Figure S5. (A) Prevalence of bovine viral diarrhea virus using nucleic acid detection methods in subgroups of adult cattle and calves. (B) Prevalence of bovine viral diarrhea virus using nucleic acid detection methods in subgroups of dairy, beef and local cattle. (C) Prevalence of bovine viral diarrhea virus detected using nucleic acid detection methods. (D) Herd level prevalence of bovine viral diarrhea virus detected using nucleic acid detection methods.
