# Supplementary material for: Comparative Analysis of the Prevalence of Bovine Viral Diarrhea Virus in Cattle Populations Based on Detection Methods: A Systematic Review and Meta-Analysis
Source: Pathogens. 2023 Aug 21;12(8):1067. doi: 10.3390/pathogens12081067 (PMC10459101; doi:10.3390/pathogens12081067)
Supplement: Supplementary file 1 [file pathogens-12-01067-s001.zip › pathogens-2545439-supplementary.pdf]

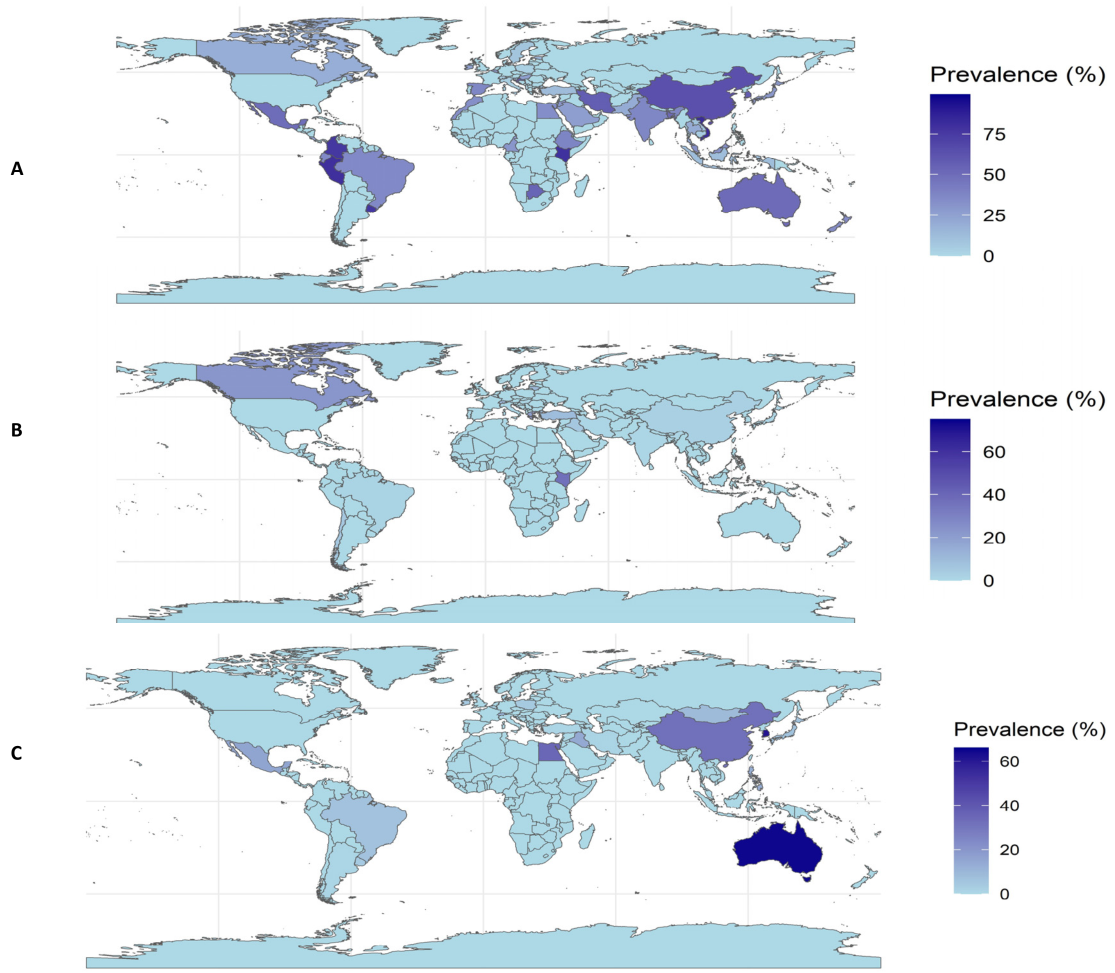

Supplementary Figure S1. Global distribution of bovine viral diarrhea virus [1-305] using **A**) antibody detection method, **B**) antigen detection methods and **C**) nucleic acid detection methods.

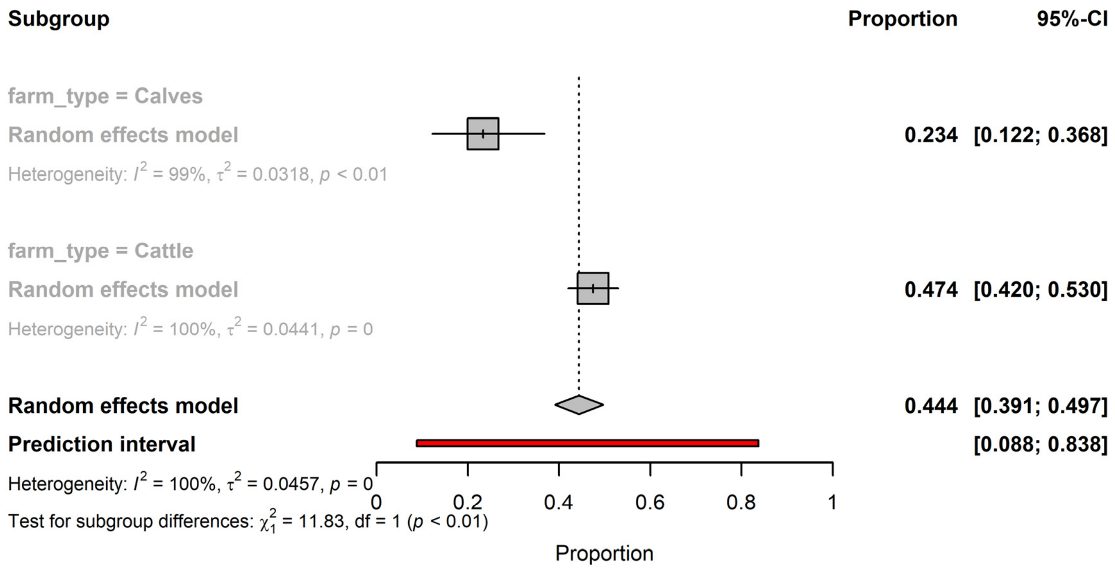

Supplementary Figure S2. A) Prevalence of bovine viral diarrhea virus using antibody detection methods in subgroups of adult cattle (cattle) and calves. To accommodate the large number of studies included in this analysis, individual studies have been masked on the subplot for optimal page fit.

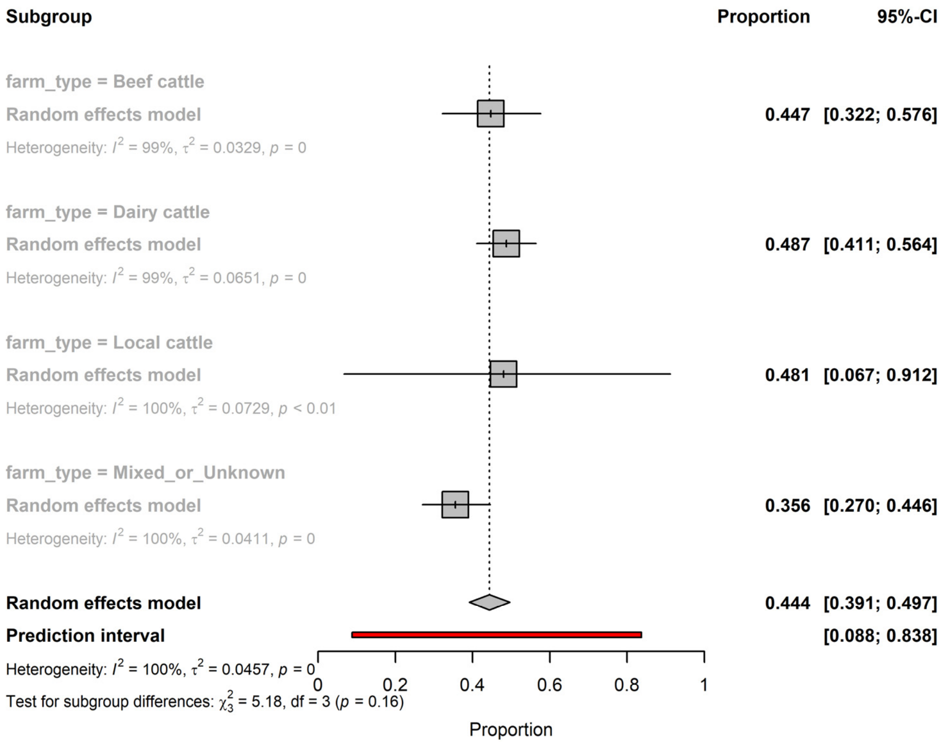

Supplementary Figure S2. **B)** Prevalence of bovine viral diarrhea virus using antibody detection methods in subgroups of Beef, dairy, local and other cattle populations. To accommodate the large number of studies included in this analysis, individual studies have been masked on the subplot for optimal page fit.

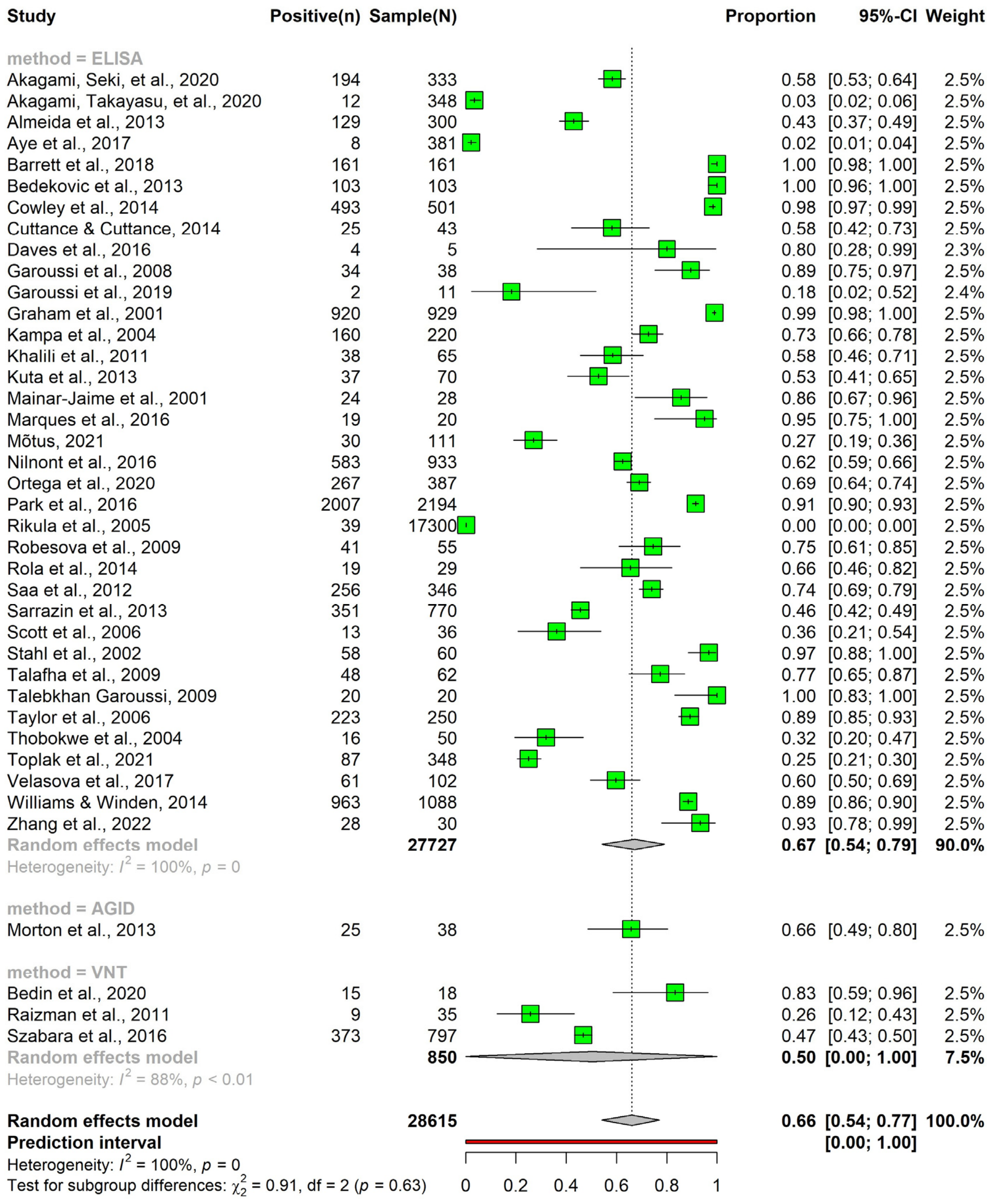

Supplementary Figure S2. **C)** Herd level prevalence of Bovine viral diarrhea virus using antibody detection methods.

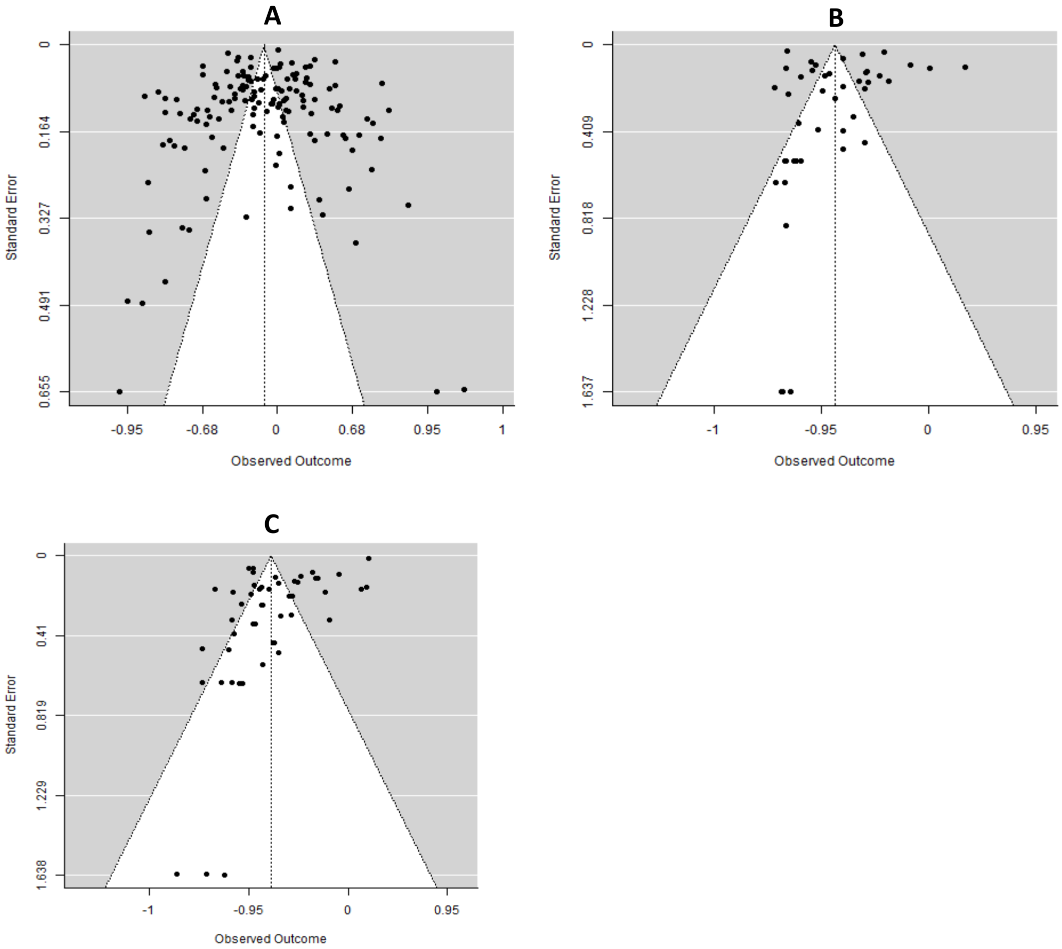

Supplementary Figure S3. Publication bias analysis using funnel plots for **A**) Antibody detection methods, **B**) Antigen detection methods and **C**) nucleic acid detection methods.

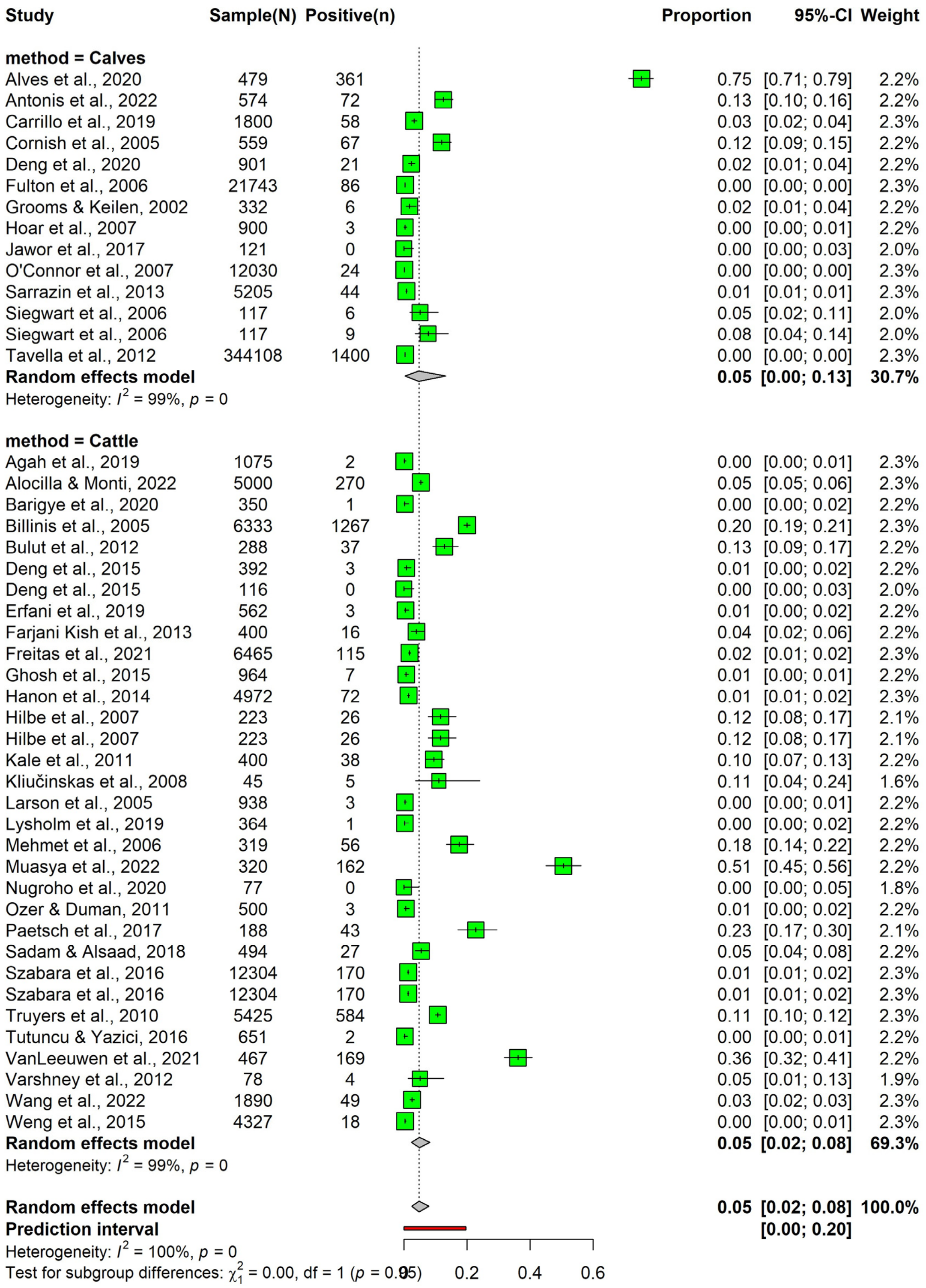

Supplementary Figure S4. A) Prevalence of Bovine viral diarrhea virus using antigen detection methods in subgroups of adult cattle and calves.

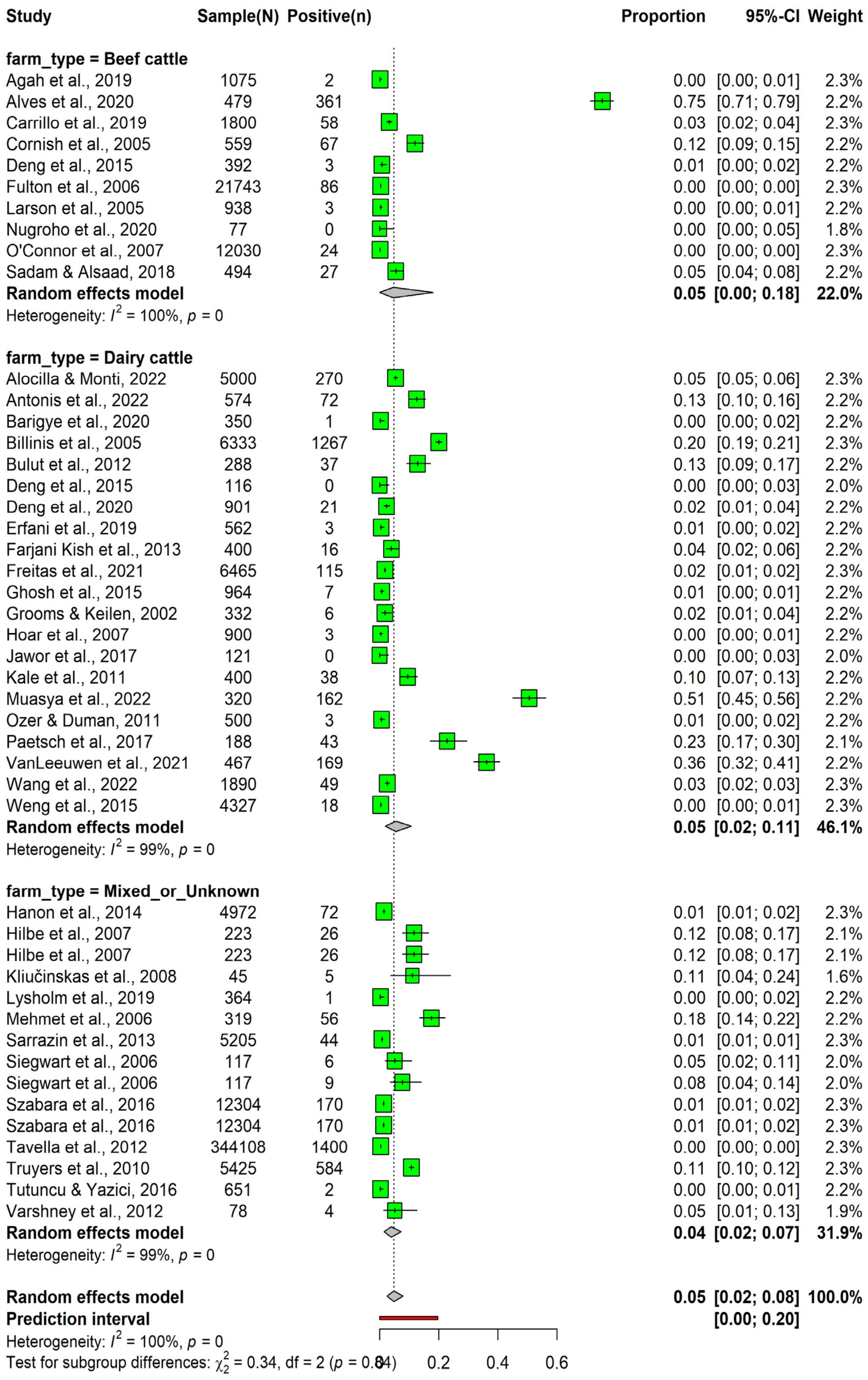

Supplementary Figure S4. **B**) Prevalence of bovine viral diarrhea virus using antigen detection methods in various farm-type subgroups.

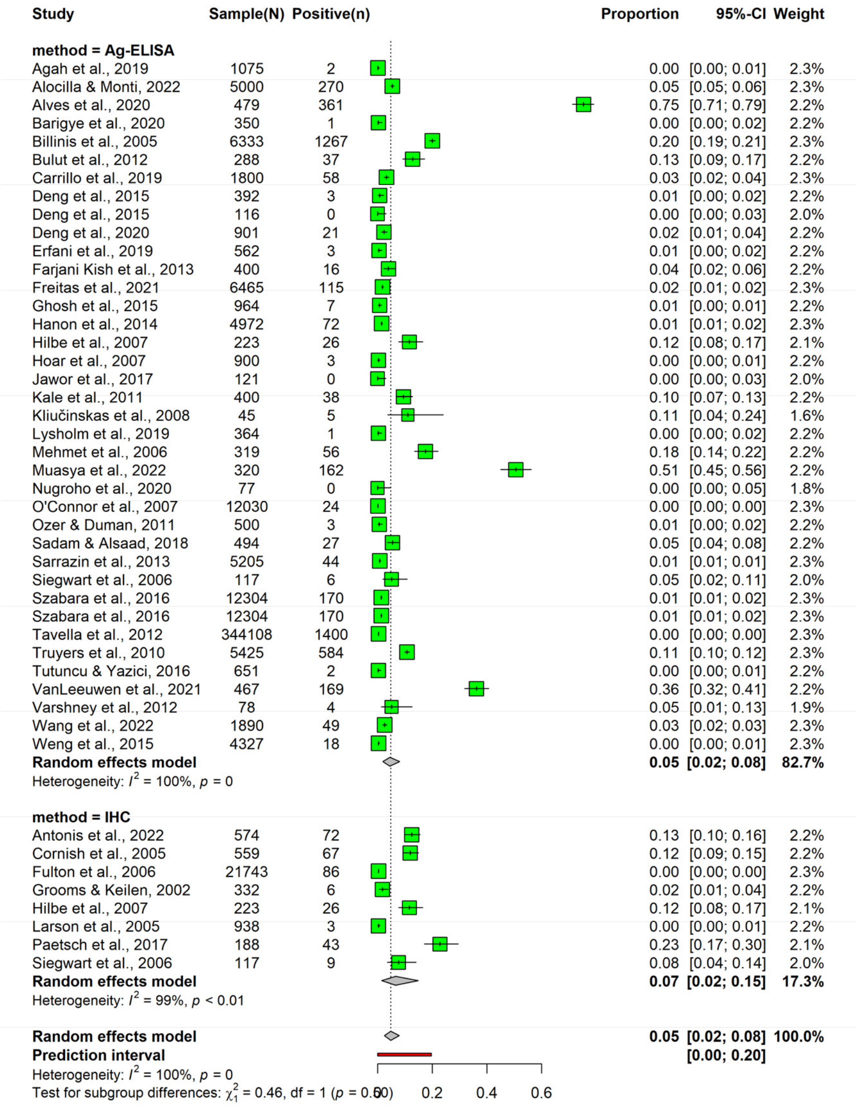

Supplementary Figure S4. **C)** Prevalence of bovine viral diarrhea virus detected using antigen ELISA and immunohistochemistry (IHC) methods.

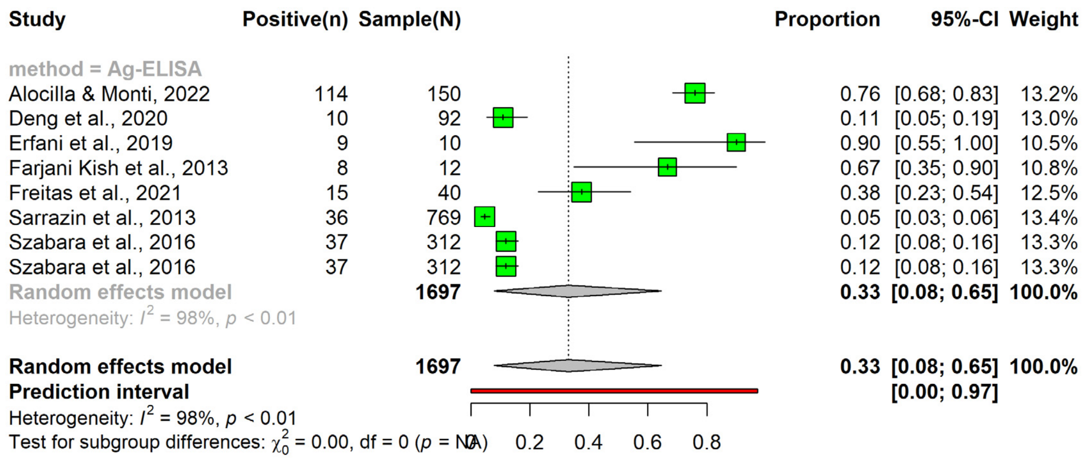

Supplementary Figure S4. **D**) Herd level prevalence of bovine viral diarrhea virus using antigen detection methods.

Study	Positive(n)	Sample(N)	Proportion	95%-CI	Weight
**method = Cattle**					
Akagami, Takayasu, et al., 2020	5	79	0.06	[0.02; 0.14]	1.8%
Assunção et al., 2022	5	77	0.06	[0.02; 0.15]	1.8%
Bedekovic et al., 2011	9	927	0.01	[0.00; 0.02]	2.1%
Behera et al., 2011	2	1446	0.00	[0.00; 0.00]	2.1%
Blakebrough-Hall et al., 2020	8	270	0.03	[0.01; 0.06]	2.1%
Byrne et al., 2017	4	2504	0.00	[0.00; 0.00]	2.2%
Chang et al., 2021	89	1234	0.07	[0.06; 0.09]	2.1%
Cirone et al., 2019	0	56	0.00	[0.00; 0.06]	1.7%
Decaro et al., 2017	16	997	0.02	[0.01; 0.03]	2.1%
Deng et al., 2015	26	200	0.13	[0.09; 0.18]	2.0%
Deng et al., 2015	42	131	0.32	[0.24; 0.41]	1.9%
El-Mohamady et al., 2020	14	40	0.35	[0.21; 0.52]	1.6%
Garoussi et al., 2019	2	138	0.01	[0.00; 0.05]	2.0%
Gomez-Romero et al., 2017	62	402	0.15	[0.12; 0.19]	2.1%
Gong et al., 2013	105	391	0.27	[0.23; 0.32]	2.1%
Hanon et al., 2014	140	4972	0.03	[0.02; 0.03]	2.2%
Hay et al., 2016	110	170	0.65	[0.57; 0.72]	2.0%
Hay et al., 2016	23235	35131	0.66	[0.66; 0.67]	0.0%
Hilbe et al., 2007	26	223	0.12	[0.08; 0.17]	2.0%
Konnai et al., 2008	12	96	0.12	[0.07; 0.21]	1.9%
Lee et al., 2008	0	1328	0.00	[0.00; 0.00]	2.1%
Maya et al., 2016	16	390	0.04	[0.02; 0.07]	2.1%
Moore et al., 2015	44	1484	0.03	[0.02; 0.04]	2.1%
Munoz-Zanzi et al., 2003	2	442	0.00	[0.00; 0.02]	2.1%
O'Neill et al., 2014	39	947	0.04	[0.03; 0.06]	2.1%
Ochirkhuu et al., 2016	11	127	0.09	[0.04; 0.15]	1.9%
Padalino et al., 2021	0	169	0.00	[0.00; 0.02]	2.0%
Peddireddi et al., 2018	174	742	0.23	[0.20; 0.27]	2.1%
Read et al., 2020	6	586	0.01	[0.00; 0.02]	2.1%
Sadam & Alsaad, 2018	69	494	0.14	[0.11; 0.17]	2.1%
Sarchet et al., 2022	105	404	0.26	[0.22; 0.31]	2.1%
Sarikaya et al., 2012	2	160	0.01	[0.00; 0.04]	2.0%
Socha et al., 2022	8	296	0.03	[0.01; 0.05]	2.1%
Spetter et al., 2021	29	2864	0.01	[0.01; 0.01]	2.2%
Toplak et al., 2021	267	9407	0.03	[0.03; 0.03]	2.2%
Zhang et al., 2022	16	385	0.04	[0.02; 0.07]	2.1%
Zhong et al., 2011	202	472	0.43	[0.38; 0.47]	2.1%
Zhou et al., 2022	258	11134	0.02	[0.02; 0.03]	2.2%
**Random effects model**		**81315**	**0.07**	**[0.04; 0.11]**	**75.5%**
Heterogeneity: I^2 = 99%, p = 0					
**method = Calves**					
Chae et al., 2021	4	544	0.01	[0.00; 0.02]	2.1%
Cho et al., 2013	2	444	0.00	[0.00; 0.02]	2.1%
Han et al., 2018	87	143	0.61	[0.52; 0.69]	2.0%
Hotchkiss et al., 2010	105	616	0.17	[0.14; 0.20]	2.1%
Martin et al., 2016	4	52	0.08	[0.02; 0.19]	1.7%
McDougall, 2021	26	1030	0.03	[0.02; 0.04]	2.1%
Rush et al., 2001	2	236	0.01	[0.00; 0.03]	2.0%
Ryu & Choi, 2019	35	635	0.06	[0.04; 0.08]	2.1%
Schefers et al., 2008	35	961	0.04	[0.03; 0.05]	2.1%
Wang et al., 2022	55	676	0.08	[0.06; 0.10]	2.1%
Weber et al., 2014	33	9078	0.00	[0.00; 0.01]	2.2%
Wernicki et al., 2015	3	78	0.04	[0.01; 0.11]	1.8%
**Random effects model**		**14493**	**0.06**	**[0.01; 0.15]**	**24.5%**
Heterogeneity: I^2 = 99%, p < 0.01					
**Random effects model**		**95808**	**0.07**	**[0.04; 0.10]**	**100.0%**
**Prediction interval**				**[0.00; 0.25]**	
Heterogeneity: I^2 = 99%, p = 0					
Test for subgroup differences: χ^2_1 = 0.06, df = 1 (p = 0.81)					

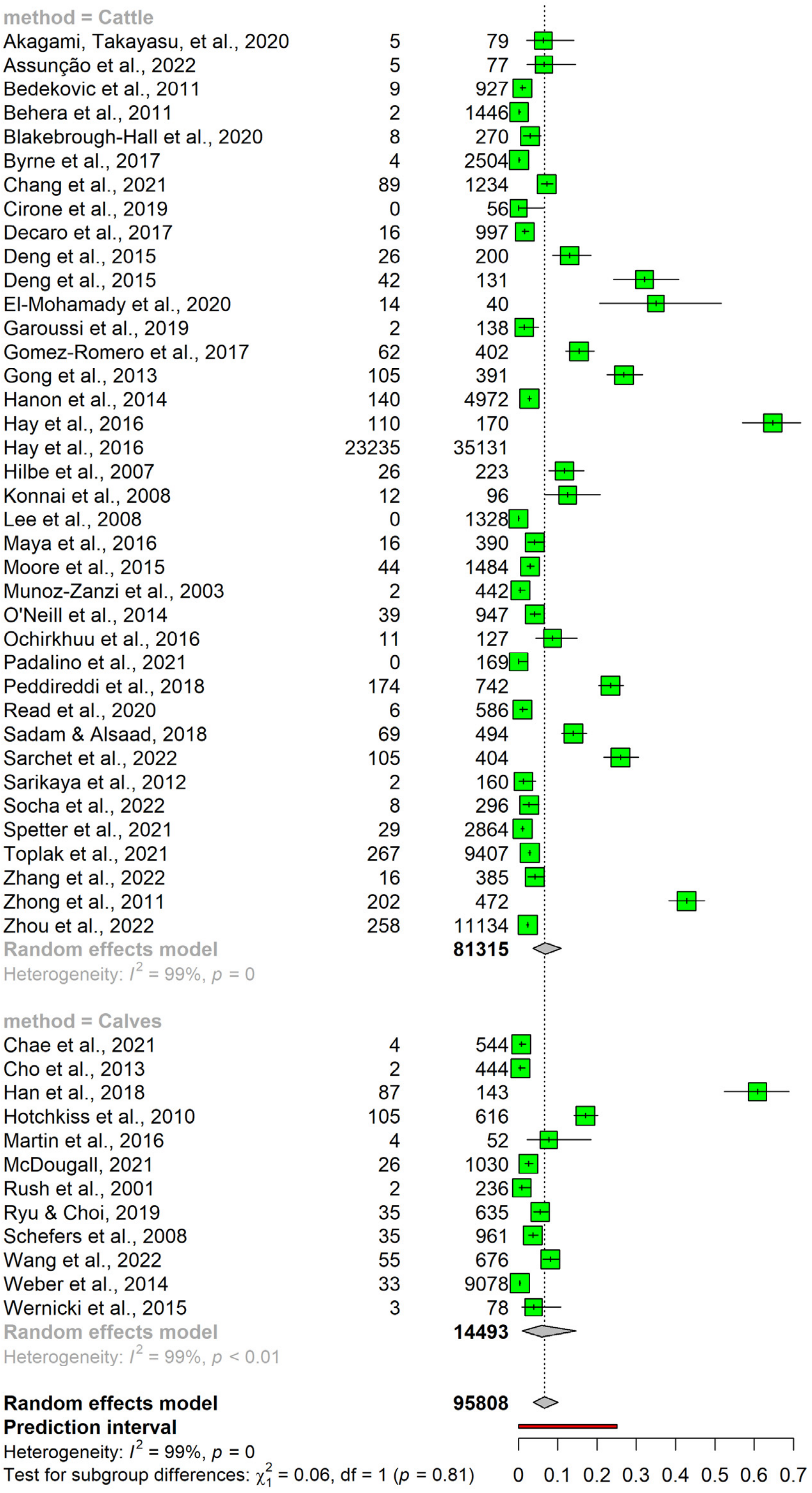

Supplementary Figure S5. A) Prevalence of bovine viral diarrhea virus using nucleic acid detection methods in subgroups of adult cattle and calves.

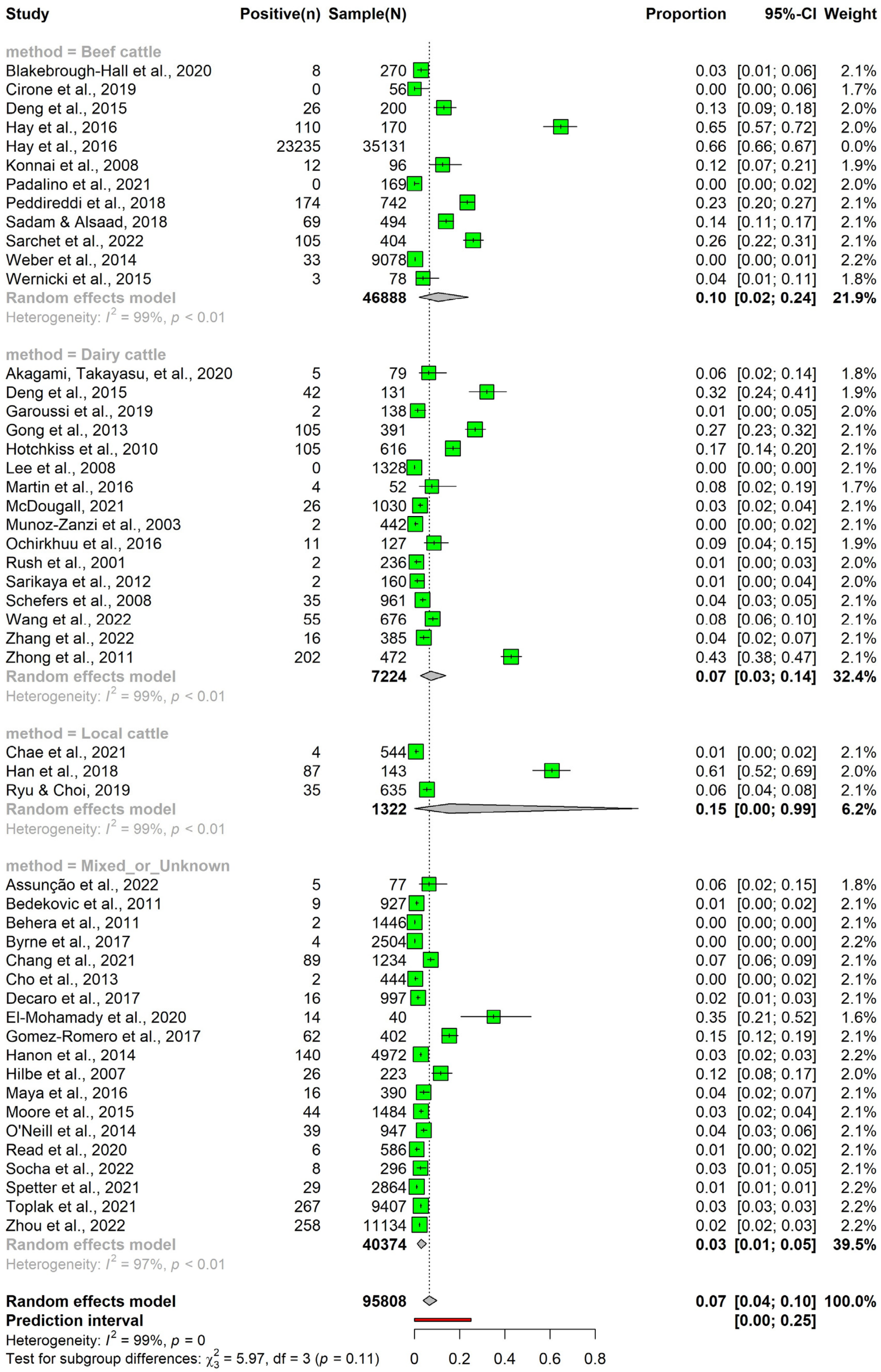

Supplementary Figure S5. B) Prevalence of bovine viral diarrhea virus using nucleic acid detection methods in subgroups of dairy, beef and local cattle.

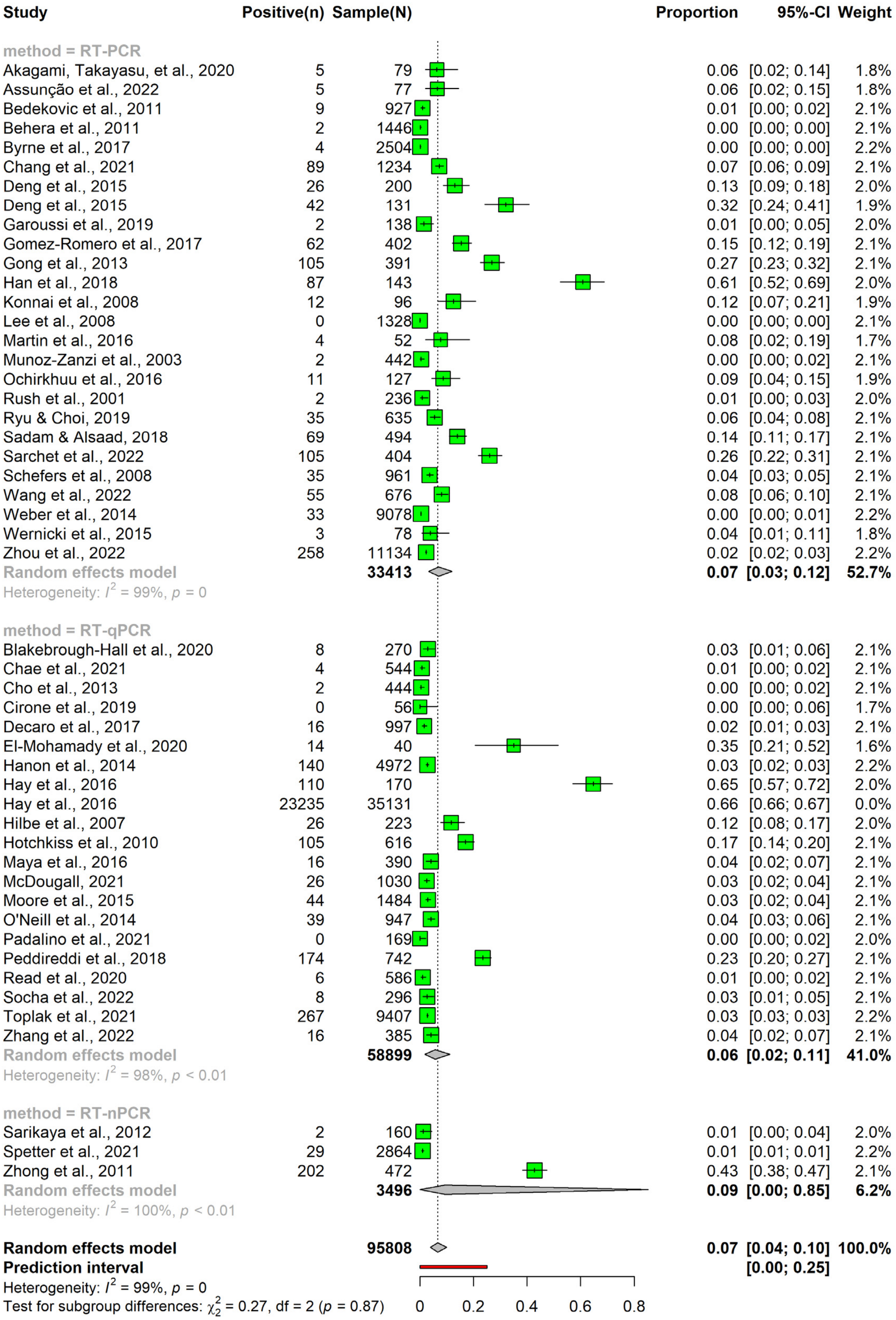

Supplementary Figure S5. C) Prevalence of bovine viral diarrhea virus detected using nucleic acid detection methods.

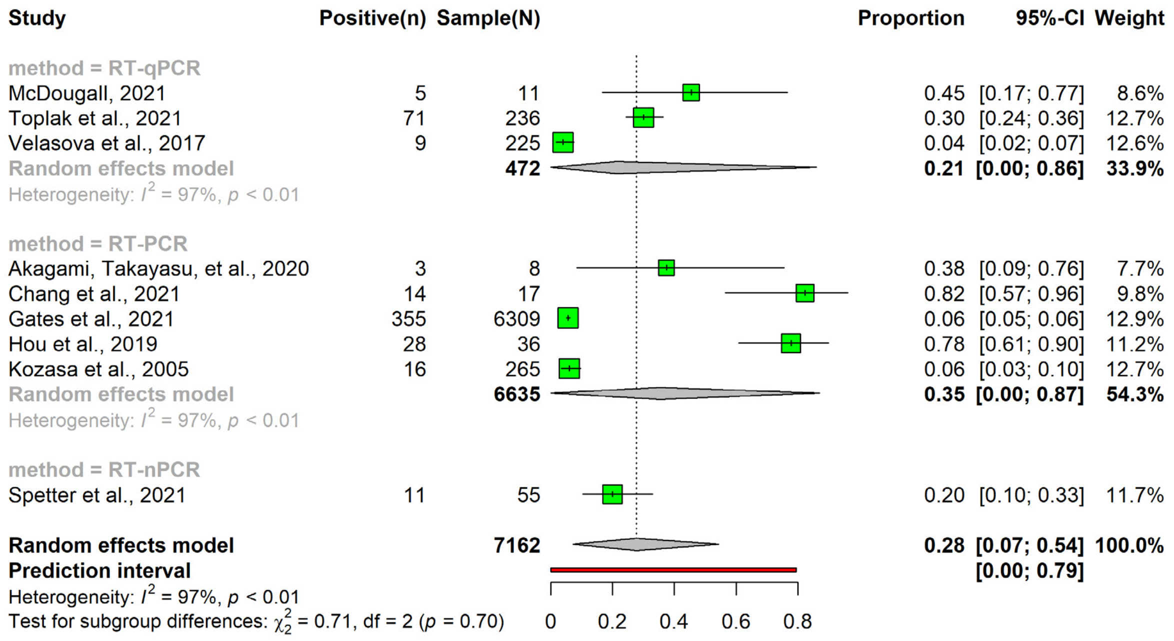

Supplementary Figure S5. **D**) Herd level prevalence of bovine viral diarrhea virus detected using nucleic acid detection methods.

Reference

1. Abuelzein, E.M.; Al-Khaliyfa, M.A.; Gameel, A.A. Natural in utero infection of neonatal calves with bovine viral diarrhoea virus on a large dairy farm in Saudi Arabia. *Onderstepoort J Vet Res* **2011**, *78*, 318, doi:10.4102/ojvr.v78i1.318.
2. Agah, M.A.; Notsu, K.; El-Khaiat, H.M.; Arikawa, G.; Kubo, M.; Mitoma, S.; Okabayashi, T.; Mekata, H.; Elhanafy, E.; El Daous, H.; et al. Slaughterhouse survey for detection of bovine viral diarrhea infection among beef cattle in Kyushu, Japan. *J Vet Med Sci* **2019**, *81*, 1450-1454, doi:10.1292/jvms.19-0045.
3. Ahmad, A.; Rabbani, M.; Muhammad, K.; Shabbir, M.Z.; Yaqub, T.; Munir, K.; Akhter, F.; Cepica, A. Prevalence of Bovine Viral Diarrhea Virus Persistency in 12 Holstein Cattle Dairy Herds of Charlottetown, Canada. *Pak J Zool* **2011**, *43*, 255-261.
4. Akagami, M.; Seki, S.; Kashima, Y.; Yamashita, K.; Oya, S.; Fujii, Y.; Takayasu, M.; Yaguchi, Y.; Suzuki, A.; Ono, Y.; et al. Risk factors associated with the within-farm transmission of bovine viral diarrhea virus and the incidence of persistently infected cattle on dairy farms from Ibaraki prefecture of Japan. *Res Vet Sci* **2020**, *129*, 187-192, doi:10.1016/j.rvsc.2020.02.001.
5. Akagami, M.; Takayasu, M.; Ooya, S.; Kashima, Y.; Tsuzuku, S.; Ootani, Y.; Ouchi, Y.; Hayama, Y. Screening of persistently infected cattle with bovine viral diarrhea virus on dairy farms by using milk tanker and bulk tank milk samples for viral RNA and viral-specific antibody detection. *J Vet Med Sci* **2020**, *82*, 607-614, doi:10.1292/jvms.19-0634.
6. AktaŞ, M.S.; ÇElİK, H. Seroprevalence of Bovine Viral Diarrhoea Virus, Bovine Herpesvirus Type 1 and Bovine Herpesvirus Type 4 infections in cattle in Ağrı Province, Eastern Anatolia Region, Turkey. *Turkish Journal of Veterinary and Animal Sciences* **2021**, *45*, 955-963, doi:10.3906/vet-2103-103.
7. Al-Khaliyfa, M.A.; Abuelzein, E.M.; Gameel, A.A. Identification of cattle persistently infected with BVDV by ear-notch testing in Saudi Arabia. *Vet Rec* **2010**, *167*, 660-661, doi:10.1136/vr.c5270.
8. Almeida, Í.C.d.; Almeida, Y.V.; Donatele, D.M.; Clipes, R.C.; Barioni, G.; Zanini, M.S.; Filippo, P.A.D. Seroprevalence and associated factors of infectious bovine rhinotracheitis and bovine

viral diarrhea in dairy cows in the Caparaó region, Espírito Santo, Brazil. *Ciência Rural* **2021**, *51*, doi:10.1590/0103-8478cr20200220.
9. Almeida, L.L.; Miranda, I.C.; Hein, H.E.; Neto, W.S.; Costa, E.F.; Marks, F.S.; Rodenbusch, C.R.; Canal, C.W.; Corbellini, L.G. Herd-level risk factors for bovine viral diarrhea virus infection in dairy herds from Southern Brazil. *Res Vet Sci* **2013**, *95*, 901-907, doi:10.1016/j.rvsc.2013.08.009.
10. Alocilla, O.A.; Monti, G. Bovine Viral Diarrhea Virus within and herd prevalence on pasture-based dairy systems, in southern Chile dairy farms. *Prev Vet Med* **2022**, *198*, 105533, doi:10.1016/j.prevetmed.2021.105533.
11. Alves, M.E.M.; Fernandes, F.D.; Monteiro, F.L.; Braunig, P.; Cargnelutti, J.F.; Flores, E.F.; Weiblen, R.; Vogel, F.S.F. Co-infection by Neopora caninum and bovine viral diarrhea virus in cattle from Rio Grande do Sul, Brazil, destined to exportation. *Pesquisa Veterinaria Brasileira* **2020**, *40*, 593-597, doi:10.1590/1678-5150-Pvb.6468.
12. Anderson, S.; Wakeley, P.; Wibberley, G.; Webster, K.; Sawyer, J. Development and evaluation of a Luminex multiplex serology assay to detect antibodies to bovine herpes virus 1, parainfluenza 3 virus, bovine viral diarrhoea virus, and bovine respiratory syncytial virus, with comparison to existing ELISA detection methods. *J Immunol Methods* **2011**, *366*, 79-88, doi:10.1016/j.jim.2011.01.010.
13. Antoci, F.; Di Bella, S.; Lupia, A.; Pennisi, M.; Salina, F.; Neve, V.C.; Coltraro, M.; Macaluso, G.; Purpari, G.; Cannella, V.; et al. Relationship between the prevalence of bovine viral diarrhea virus and animal welfare assessment, serum cortisol levels and hematological parameters in calves from three different Sicilian farms. *Large Animal Review* **2022**, *28*, 111-116.
14. Antonis, A.F.G.; Swanenburg, M.; Wisselink, H.J.; Smid, B.; van Klink, E.; Hagenaars, T.J. Respiratory pathogens in veal calves: Inventory of circulating pathogens. *Vet Microbiol* **2022**, *274*, 109571, doi:10.1016/j.vetmic.2022.109571.
15. Aragaw, K.; Regassa, F.; Sibhat, B.; Abayneh, T.; Gelaye, E.; Deresse, G.; Egan, S.; Asmare, K. Seroprevalence and association of bovine viral diarrhea virus (BVDV) serostatus with reproductive problems in dairy cattle in central and southern Ethiopia. *Trop Anim Health Prod* **2021**, *53*, 471, doi:10.1007/s11250-021-02904-y.
16. Aragaw, K.; Sibhat, B.; Ayelet, G.; Skjerve, E.; Gebremedhin, E.Z.; Asmare, K. Seroprevalence and factors associated with bovine viral diarrhea virus (BVDV) infection in dairy cattle in three milksheds in Ethiopia. *Trop Anim Health Prod* **2018**, *50*, 1821-1827, doi:10.1007/s11250-018-1624-5.
17. Arbulú-García, C.; Morales-Cauti, S. Seroprevalencia de anticuerpos contra el virus de la diarrea viral bovina en bovinos de crianza extensiva en tres distritos de Ayacucho, Perú. *Revista de Investigaciones Veterinarias del Perú* **2021**, *32*, doi:10.15381/rivep.v32i3.20401.
18. Aslan, M.E.; Azkur, A.K.; Gazyagci, S. Epidemiology and genetic characterization of BVDV, BHV-1, BHV-4, BHV-5 and Brucella spp. infections in cattle in Turkey. *J Vet Med Sci* **2015**, *77*, 1371-1377, doi:10.1292/jvms.14-0657.
19. Asmare, K.; Regassa, F.; Robertson, L.J.; Martin, A.D.; Skjerve, E. Reproductive disorders in relation to Neospora caninum, Brucella spp. and bovine viral diarrhoea virus serostatus in breeding and dairy farms of central and southern Ethiopia. *Epidemiol Infect* **2013**, *141*, 1772-1780, doi:10.1017/S0950268812002191.
20. Assunção, S.F.V.; Antos, A.; Barbosa, J.D.; Reis, J.K.P.; Larska, M.; Oliveira, C.H.S. Diagnosis and phylogenetic analysis of bovine viral diarrhea virus in cattle (Bos taurus) and buffaloes (Bubalus bubalis) from the Amazon region and Southeast Brazil. *Pesquisa Veterinária Brasileira* **2022**, *42*, doi:10.1590/1678-5150-pvb-6955.
21. Aye, Y.M.; Aung, M.; Kyaw, W.O.; Naing, T.; Po, S.P. Prevalence and associated factors with bovine viral diarrhoea virus antibodies in the bulk tank milk of small scale dairy herds in central Myanmar. *Advances in Animal and Veterinary Sciences* **2017**, *5*, 316-323, doi:10.17582/journal.aavs/2017/5.8.316.323.

22. Barigye, R.; Hassan, N.A.; AlQubaisi, D.M.N.; Abdalla-Alfaki, I.M. Serological evidence of Coxiella burnetii, Leptospira interrogans Hardjo, Neospora caninum and bovine pestivirus infections in a dairy cattle herd from the United Arab Emirates. *Vet Ital* **2020**, *56*, 163-168, doi:10.12834/VetIt.2257.12932.1.
23. Barrett, D.; Clarke, A.; O'Keeffe, K.; Kellegher, P.; Comerford, J.; Lane, E.; Byrne, A.W. BVD seroprevalence in the Irish cattle population as the national BVD programme progresses toward eradication. *Bmc Vet Res* **2022**, *18*, 210, doi:10.1186/s12917-022-03318-0.
24. Barrett, D.; Parr, M.; Fagan, J.; Johnson, A.; Tratalos, J.; Lively, F.; Diskin, M.; Kenny, D. Prevalence of Bovine Viral Diarrhoea Virus (BVDV), Bovine Herpes Virus 1 (BHV 1), Leptospirosis and Neosporosis, and associated risk factors in 161 Irish beef herds. *Bmc Vet Res* **2018**, *14*, 8, doi:10.1186/s12917-017-1324-9.
25. Basqueira, N.S.; Martin, C.C.; Costa, J.F.D.; Okuda, L.H.; Pituco, M.E.; Batista, C.F.; Della Libera, A.M.M.P.; Gomes, V. Bovine Respiratory Disease (BRD) Complex as a Signal for Bovine Viral Diarrhea Virus (BVDV) Presence in the Herd. *Acta Scientiae Veterinariae* **2017**, *45*, 6.
26. Bedeković, T.; Lemo, N.; Barbić, L.; Cvetnić, Ž.; Lojkić, I.; Benić, M.; Čač, Ž.; Lojkić, M.; Madić, J. Influence of category, herd size, grazing and management on epidemiology of bovine viral diarrhoea in dairy herds. *Acta Veterinaria Brno* **2013**, *82*, 125-130, doi:10.2754/avb201382020125.
27. Bedekovic, T.; Lemo, N.; Lojkic, I.; Cvetnic, Z.; Cac, Z.; Madic, J. Development of an indirect immunofluorescence assay for diagnosis of bovine viral diarrhoea virus on ear notch tissue samples in cattle infected persistently. *J Virol Methods* **2011**, *178*, 59-62, doi:10.1016/j.jviromet.2011.08.012.
28. Bedekovic, T.; Mihaljevic, Z.; Jungic, A.; Lemo, N.; Lojkic, I.; Cvetnic, Z.; Cac, Z. Detection of Bovine viral diarrhea virus-specific neutralizing antibodies in fresh colostrum: a modification of the virus neutralization test. *J Vet Diagn Invest* **2013**, *25*, 263-266, doi:10.1177/1040638713478813.
29. Bedin, A.; Sott, T.; Freitas, F.L.d.C.; Nascif Junior, I.A.; Rozolen, J.M.; Fonseca-Alves, C.E.; Elias, F. Serological prevalence of bovine viral diarrhoea (BVD-1) in non-vaccinated dairy cattle in the municipality of Realeza-Paraná. *Semina: Ciências Agrárias* **2020**, *41*, 907-914, doi:10.5433/1679-0359.2020v41n3p907.
30. Behera, S.P.; Mishra, N.; Vilcek, S.; Rajukumar, K.; Nema, R.K.; Prakash, A.; Kalaiyarasu, S.; Dubey, S.C. Genetic and antigenic characterization of bovine viral diarrhoea virus type 2 isolated from cattle in India. *Comp Immunol Microbiol Infect Dis* **2011**, *34*, 189-196, doi:10.1016/j.cimid.2010.11.002.
31. Bezerra, N.P.C.; Bezerra, D.C.; Santos, H.P.; Pereira, H.d.M.; Silva, A.L.A. Risk factors analysis applied to antibodies to Bovine Herpesvirus Type 1, Bovine Viral Diarrhea Virus, Bovine Leukemia Virus and Brucella abortus among cattle: a cross-sectional study. *Acta Veterinaria Brasilica* **2019**, *13*, 5-12, doi:10.21708/avb.2019.13.1.7818.
32. Bilge-Dagalp, S.; Can-Sahna, K.; Yildirm, Y.; Karaoglu, T.; Alkan, F.; Burgu, I. Effects of bovine leucosis virus (BLV) infection on the bovine viral diarrhoea virus (BVDV) and bovine herpes virus 1 (BHV1) seroprevalences in dairy herds in Turkey. *Revue De Medecine Veterinaire* **2008**, *159*, 385-390.
33. Billinis, C.; Leontides, L.; Amiridis, G.S.; Spyrou, V.; Kostoulas, P.; Sofia, M. Prevalence of BVDV infection in Greek dairy herds. *Prev Vet Med* **2005**, *72*, 75-79; discussion 215-219, doi:10.1016/j.prevetmed.2005.05.011.
34. Bjorkman, C.; Alenius, S.; Manuelsson, U.; Uggla, A. Neospora caninum and bovine virus diarrhoea virus infections in Swedish dairy cows in relation to abortion. *Vet J* **2000**, *159*, 201-206, doi:10.1053/tvjl.1999.0446.
35. Blakebrough-Hall, C.; Hick, P.; Gonzalez, L.A. Predicting bovine respiratory disease outcome in feedlot cattle using latent class analysis. *J Anim Sci* **2020**, *98*, doi:10.1093/jas/skaa381.

36. Blakebrough-Hall, C.; Hick, P.; Mahony, T.J.; Gonzalez, L.A. Factors associated with bovine respiratory disease case fatality in feedlot cattle. *J Anim Sci* **2022**, *100*, doi:10.1093/jas/skab361.
37. Bulut, O.; Avci, O.; Yapici, O.; Yavru, S.; Simsek, A. Serological and virological investigation of bovine viral diarrhea virus (BVDV) infection in cattle with abortion problem. *Febs J* **2012**, *279*, 573-573.
38. Byrne, A.W.; Graham, J.; Brown, C.; Donaghy, A.; Guelbenzu-Gonzalo, M.; McNair, J.; Skuce, R.; Allen, A.; McDowell, S. Bovine tuberculosis visible lesions in cattle culled during herd breakdowns: the effects of individual characteristics, trade movement and co-infection. *Bmc Vet Res* **2017**, *13*, 400, doi:10.1186/s12917-017-1321-z.
39. Callaby, R.; Toye, P.; Jennings, A.; Thumbi, S.M.; Coetzer, J.A.; Conradie Van Wyk, I.C.; Hanotte, O.; Mbole-Kariuki, M.N.; Bronsvoort, B.M.; Kruuk, L.E.; et al. Seroprevalence of respiratory viral pathogens of indigenous calves in Western Kenya. *Res Vet Sci* **2016**, *108*, 120-124, doi:10.1016/j.rvsc.2016.08.010.
40. Carrillo, H.A.M.; Silveira, S.R.B.; Miranda, A.S.; Rodrigues, É.D.L.; Salvarani, F.M. Prevalence of bovine brucellosis, paratuberculosis, enzootic leucosis, and antigen-reactive agents to bovine viral diarrhea virus in animals up to one year old. *Semina: Ciências Agrárias* **2019**, *40*, 485-489, doi:10.5433/1679-0359.2019v40n1p485.
41. Chae, J.B.; Kim, H.C.; Kang, J.G.; Choi, K.S.; Chae, J.S.; Yu, D.H.; Park, B.K.; Oh, Y.S.; Choi, H.J.; Park, J. The prevalence of causative agents of calf diarrhea in Korean native calves. *J Anim Sci Technol* **2021**, *63*, 864-871, doi:10.5187/jast.2021.e63.
42. Chang, L.; Qi, Y.; Liu, D.; Du, Q.; Zhao, X.; Tong, D. Molecular detection and genotyping of bovine viral diarrhea virus in Western China. *Bmc Vet Res* **2021**, *17*, 66, doi:10.1186/s12917-021-02747-7.
43. Cho, Y.I.; Han, J.I.; Wang, C.; Cooper, V.; Schwartz, K.; Engelken, T.; Yoon, K.J. Case-control study of microbiological etiology associated with calf diarrhea. *Vet Microbiol* **2013**, *166*, 375-385, doi:10.1016/j.vetmic.2013.07.001.
44. Cirone, F.; Padalino, B.; Tullio, D.; Capozza, P.; Lo Surdo, M.; Lanave, G.; Pratelli, A. Prevalence of Pathogens Related to Bovine Respiratory Disease Before and After Transportation in Beef Steers: Preliminary Results. *Animals (Basel)* **2019**, *9*, doi:10.3390/ani9121093.
45. Cornish, T.E.; van Olphen, A.L.; Cavender, J.L.; Edwards, J.M.; Jaeger, P.T.; Vieyra, L.L.; Woodard, L.F.; Miller, D.R.; O'Toole, D. Comparison of ear notch immunohistochemistry, ear notch antigen-capture ELISA, and buffy coat virus isolation for detection of calves persistently infected with bovine viral diarrhea virus. *J Vet Diagn Invest* **2005**, *17*, 110-117, doi:10.1177/104063870501700203.
46. Cowley, D.J.; Graham, D.A.; Guelbenzu, M.; Doherty, M.L.; More, S.J. Aspects of bovine herpesvirus 1 and bovine viral diarrhoea virus herd-level seroprevalence and vaccination in dairy and beef herds in Northern Ireland. *Ir Vet J* **2014**, *67*, 18, doi:10.1186/2046-0481-67-18.
47. Cusack, P.; Bergman, E.L.; Hay, K.E.; Morton, J.M. Serological status of cattle to bovine viral diarrhoea virus and bovine herpesvirus 1 at entry to and exit from Australian feedlot backgrounding facilities. *Aust Vet J* **2021**, *99*, 20-23, doi:10.1111/avj.13021.
48. Cuttance, W.G.; Cuttance, E.L. Analysis of individual farm investigations into bovine viral diarrhoea in beef herds in the North Island of New Zealand. *N Z Vet J* **2014**, *62*, 338-342, doi:10.1080/00480169.2014.928925.
49. Daly, R.F.; Neiger, R.D. Outbreak of Salmonella enterica serotype Newport in a beef cow-calf herd associated with exposure to bovine viral diarrhea virus. *J Am Vet Med Assoc* **2008**, *233*, 618-623, doi:10.2460/javma.233.4.618.
50. Daves, L.; Yimer, N.; Arshad, S.S.; Sarsaifi, K.; Omar, M.A.; Yusoff, R.; Haron, A.W.; Abdullah, F.F.J. Seroprevalence of Bovine Viral Diarrhea Virus Infection and Associated Risk Factors in Cattle in Selangor, Malaysia. *Veterinary Medicine - Open Journal* **2016**, *1*, 22-28, doi:10.17140/vmoj-1-105.

51. de Melo, C.B.; Leite, R.C.; Lobato, Z.I.; Leite, R.C. Infection by Neospora caninum associated with bovine herpesvirus 1 and bovine viral diarrhea virus in cattle from Minas Gerais State, Brazil. *Vet Parasitol* **2004**, *119*, 97-105, doi:10.1016/j.vetpar.2003.12.002.
52. Decaro, N.; Lucente, M.S.; Lanave, G.; Gargano, P.; Larocca, V.; Losurdo, M.; Ciambrone, L.; Marino, P.A.; Parisi, A.; Casalinuovo, F.; et al. Evidence for Circulation of Bovine Viral Diarrhoea Virus Type 2c in Ruminants in Southern Italy. *Transbound Emerg Dis* **2017**, *64*, 1935-1944, doi:10.1111/tbed.12592.
53. Demil, E.; Fentie, T.; Vidal, G.; Jackson, W.; Lane, J.; Mekonnen, S.A.; Smith, W. Prevalence of bovine viral diarrhea virus antibodies and risk factors in dairy cattle in Gondar city, Northwest Ethiopia. *Prev Vet Med* **2021**, *191*, 105363, doi:10.1016/j.prevetmed.2021.105363.
54. Deng, J.F.R.H.C.C.G. Prevalence study and genetic typing of bovine viral diarrhea virus (BVDV) in four bovine species in China. *PloS one* **2015**.
55. Deng, M.; Chen, N.; Guidarini, C.; Xu, Z.; Zhang, J.; Cai, L.; Yuan, S.; Sun, Y.; Metcalfe, L. Prevalence and genetic diversity of bovine viral diarrhea virus in dairy herds of China. *Vet Microbiol* **2020**, *242*, 108565, doi:10.1016/j.vetmic.2019.108565.
56. Deng, M.; Ji, S.; Fei, W.; Raza, S.; He, C.; Chen, Y.; Chen, H.; Guo, A. Prevalence study and genetic typing of bovine viral diarrhea virus (BVDV) in four bovine species in China. *PLoS One* **2015**, *10*, e0121718, doi:10.1371/journal.pone.0121718.
57. Diéguez, F.J.; Yus, E.; Sanjuán, M.L.; Vilar, M.J.; Arnaiz, I. Monitoring bovine viral diarrhea virus (BVDV) infection status in dairy herds. *Pesquisa Veterinária Brasileira* **2008**, *28*, 588-592, doi:10.1590/s0100-736x2008001200004.
58. Dimitrova, E.; Martinov, S. Study on Prevalence of Bovine Viral Diarrhea Virus (BVDV) Antibodies in 19 Bulgarian Dairy Cattle and Sheep Herds with Reproductive Problems. *Biotechnol Biotec Eq* **2014**, *13*, 51-54, doi:10.1080/13102818.1999.10819039.
59. Duman, R.; Yavru, S.; Kale, M.; Avci, O. Seroprevalence of Viral Upper Respiratory Infections in Dairy Cattle. *Kafkas Universitesi Veteriner Fakultesi Dergisi* **2009**, *15*, 539-542.
60. Duman, R.; Yavru, S.; Kale, M.; Avci, O. Süt Sığırlarında Viral Üst Solunum Yolu Enfeksiyonlarının Seroprevalansı. *Kafkas Universitesi Veteriner Fakultesi Dergisi* **2009**, doi:10.9775/kvfd.2009.051-A.
61. Duong, M.C.; Alenius, S.; Huong, L.T.; Bjorkman, C. Prevalence of Neospora caninum and bovine viral diarrhoea virus in dairy cows in Southern Vietnam. *Vet J* **2008**, *175*, 390-394, doi:10.1016/j.tvjl.2006.01.016.
62. El-Mohamady, R.S.; Behour, T.S.; Rawash, Z.M. Concurrent detection of bovine viral diarrhoea virus and bovine herpesvirus-1 in bulls' semen and their effect on semen quality. *Int J Vet Sci Med* **2020**, *8*, 106-114, doi:10.1080/23144599.2020.1850197.
63. Elhassan, A.M.; Fadol, M.A.; El-Hussein, A.M. Seroprevalence of Bovine Herpes Virus-1, Bovine Herpes Virus-4 and Bovine Viral Diarrhea Virus in Dairy Cattle in Sudan. *Pak Vet J* **2011**, *31*, 317-320.
64. Erfani, A.M.; Bakhshesh, M.; Fallah, M.H.; Hashemi, M. Seroprevalence and risk factors associated with bovine viral diarrhea virus and bovine herpes virus-1 in Zanjan Province, Iran. *Trop Anim Health Prod* **2019**, *51*, 313-319, doi:10.1007/s11250-018-1687-3.
65. Evans, C.A.; Cockcroft, P.D.; Reichel, M.P. Antibodies to bovine viral diarrhoea virus (BVDV) in water buffalo (Bubalus bubalis) and cattle from the Northern Territory of Australia. *Aust Vet J* **2016**, *94*, 423-426, doi:10.1111/avj.12517.
66. Falkenberg, S.M.; Bauermann, F.V.; Scoles, G.A.; Bonilla, D.; Dassanayake, R.P. A Serosurvey for Ruminant Pestivirus Exposure Conducted Using Sera From Stray Mexico Origin Cattle Captured Crossing Into Southern Texas. *Front Vet Sci* **2022**, *9*, 821247, doi:10.3389/fvets.2022.821247.
67. Fanelli, A.; Cirilli, M.; Lucente, M.S.; Zarea, A.A.K.; Buonavoglia, D.; Tempesta, M.; Greco, G. Fatal Calf Pneumonia Outbreaks in Italian Dairy Herds Involving Mycoplasma bovis and Other Agents of BRD Complex. *Front Vet Sci* **2021**, *8*, 742785, doi:10.3389/fvets.2021.742785.

68. Farjani Kish, G.; Khodakaram-Tafti, A.; Mohammadi, A. Serological survey of bovine viral diarrhoea virus by antigen capture ELISA in dairy herds in Fars Province, Iran. *Bulgarian Journal of Veterinary Medicine* **2013**, *16*, 217-222.
69. Fernandes, L.G.; Nogueira, A.H.; De Stefano, E.; Pituco, E.M.; Ribeiro, C.P.; Alves, C.J.; Oliveira, T.S.; Clementino, I.J.; de Azevedo, S.S. Herd-level prevalence and risk factors for bovine viral diarrhea virus infection in cattle in the State of Paraiba, Northeastern Brazil. *Trop Anim Health Prod* **2016**, *48*, 157-165, doi:10.1007/s11250-015-0937-x.
70. Fernandes, L.G.; Pituco, E.M.; de Campos Nogueira Romaldini, A.H.; De Stefano, E.; Clementino, I.J.; Maia, A.R.A.; de Sousa Americo Batista Santos, C.; Alves, C.J.; de Azevedo, S.S. Spatial analysis for bovine viral diarrhea virus and bovine herpesvirus type 1 infections in the state of Paraiba, northeastern Brazil. *Bmc Vet Res* **2018**, *14*, 102, doi:10.1186/s12917-018-1412-5.
71. Foddai, A.; Enoe, C.; Stockmarr, A.; Krogh, K.; Uttenthal, A. Challenges for bovine viral diarrhoea virus antibody detection in bulk milk by antibody enzyme-linked immunosorbent assays due to changes in milk production levels. *Acta Vet Scand* **2015**, *57*, 32, doi:10.1186/s13028-015-0125-z.
72. Freitas, B.B.; Correa, A.M.F.; Valotto, A.A.; Marcom, N.N.; Paulino, L.R.; Brum, J.S.; Perotta, J.H.; Barros Filho, I.R. Prevalence of bovines persistently infected with bovine viral diarrhea virus (BVDV) in dairy cattle herds in Paraná State, Brazil. *Pesquisa Veterinária Brasileira* **2021**, *41*, doi:10.1590/1678-5150-pvb-6622.
73. Fulton, R.W.; Blood, K.S.; Panciera, R.J.; Payton, M.E.; Ridpath, J.F.; Confer, A.W.; Saliki, J.T.; Burge, L.T.; Welsh, R.D.; Johnson, B.J.; et al. Lung pathology and infectious agents in fatal feedlot pneumonias and relationship with mortality, disease onset, and treatments. *J Vet Diagn Invest* **2009**, *21*, 464-477, doi:10.1177/104063870902100407.
74. Fulton, R.W.; Hessman, B.; Johnson, B.J.; Ridpath, J.F.; Saliki, J.T.; Burge, L.J.; Sjeklocha, D.; Confer, A.W.; Funk, R.A.; Payton, M.E. Evaluation of diagnostic tests used for detection of bovine viral diarrhea virus and prevalence of subtypes 1a, 1b, and 2a in persistently infected cattle entering a feedlot. *J Am Vet Med Assoc* **2006**, *228*, 578-584, doi:10.2460/javma.228.4.578.
75. Fulton, R.W.; Purdy, C.W.; Confer, A.W.; Saliki, J.T.; Loan, R.W.; Briggs, R.E.; Burge, L.J. Bovine viral diarrhea viral infections in feeder calves with respiratory disease: interactions with Pasteurella spp., parainfluenza-3 virus, and bovine respiratory syncytial virus. *Can J Vet Res* **2000**, *64*, 151-159.
76. Fulton, R.W.; Whitley, E.M.; Johnson, B.J.; Ridpath, J.F.; Kapil, S.; Burge, L.J.; Cook, B.J.; Confer, A.W. Prevalence of bovine viral diarrhea virus (BVDV) in persistently infected cattle and BVDV subtypes in affected cattle in beef herds in south central United States. *Can J Vet Res* **2009**, *73*, 283-291.
77. Gaeta, N.C.; Ribeiro, B.L.M.; Alemán, M.A.R.; Yoshihara, E.; Marques, E.C.; Hellmeister, A.N.; Pituco, E.M.; Gregory, L. Serological investigation of antibodies against respiratory viruses in calves from Brazilian family farming and their relation to clinical signs of bovine respiratory disease. *Pesquisa Veterinária Brasileira* **2018**, *38*, 642-648, doi:10.1590/1678-5150-pvb-5234.
78. Gagea, M.I.; Bateman, K.G.; Shanahan, R.A.; van Dreumel, T.; McEwen, B.J.; Carman, S.; Archambault, M.; Caswell, J.L. Naturally occurring Mycoplasma bovis-associated pneumonia and polyarthritis in feedlot beef calves. *J Vet Diagn Invest* **2006**, *18*, 29-40, doi:10.1177/104063870601800105.
79. Gagea, M.I.; Bateman, K.G.; van Dreumel, T.; McEwen, B.J.; Carman, S.; Archambault, M.; Shanahan, R.A.; Caswell, J.L. Diseases and pathogens associated with mortality in Ontario beef feedlots. *J Vet Diagn Invest* **2006**, *18*, 18-28, doi:10.1177/104063870601800104.
80. García, A.A.; Torreglosa, J.T.; Marín, D.D.; Bernal, M.K.M.; Rolim Filho, S.T.; Pereira, W.L.A. Leptospirosis, bovine viral diarrhea and infectious bovine rhinotracheitis: prevalence in

Colombian cattle and buffaloes. *Acta Scientiarum. Animal Sciences* **2022**, *44*, doi:10.4025/actascianimsci.v44i1.54875.
81. Garoussi, M.T.; Haghparast, A.; Estajee, H. Prevalence of bovine viral diarrhoea virus antibodies in bulk tank milk of industrial dairy cattle herds in suburb of Mashhad-Iran. *Prev Vet Med* **2008**, *84*, 171-176, doi:10.1016/j.prevetmed.2007.12.016.
82. Garoussi, M.T.; Mehrzad, J.; Nejati, A. Investigation of persistent infection of bovine viral diarrhea virus (BVDV) in Holstein dairy cows. *Trop Anim Health Prod* **2019**, *51*, 853-858, doi:10.1007/s11250-018-1765-6.
83. Gates, M.C.; Evans, C.A.; Han, J.H.; Heuer, C.; Weston, J.F. Practices and opinions of New Zealand beef cattle farmers towards bovine viral diarrhoea control in relation to real and perceived herd serological status. *N Z Vet J* **2020**, *68*, 92-100, doi:10.1080/00480169.2019.1692735.
84. Gates, M.C.; Evans, C.A.; Heuer, C.; Voges, H.; Weston, J.F. Temporal trends in bulk tank milk antibody ELISA and PCR test results for bovine viral diarrhoea in New Zealand pastoral dairy herds. *N Z Vet J* **2021**, *69*, 73-82, doi:10.1080/00480169.2020.1806756.
85. Gates, M.C.; Woolhouse, M.E.; Gunn, G.J.; Humphry, R.W. Relative associations of cattle movements, local spread, and biosecurity with bovine viral diarrhoea virus (BVDV) seropositivity in beef and dairy herds. *Prev Vet Med* **2013**, *112*, 285-295, doi:10.1016/j.prevetmed.2013.07.017.
86. Ghosh, R.; Chowdhury, S.; Mitra, J.; Sarkar, S.N.; Batabyal, S. Prevalence of Bovine Viral Diarrhoea Virus in West Bengal, India. *Explor Anim Med Res* **2015**, *5*, 152-159.
87. Gomez-Romero, N.; Basurto-Alcantara, F.J.; Verdugo-Rodriguez, A.; Bauermann, F.V.; Ridpath, J.F. Genetic diversity of bovine viral diarrhea virus in cattle from Mexico. *J Vet Diagn Invest* **2017**, *29*, 362-365, doi:10.1177/1040638717690187.
88. Gong, X.; Cao, X.; Zheng, F.; Chen, Q.; Zhou, J.; Yin, H.; Liu, L.; Cai, X. Identification and characterization of a novel subgenotype of bovine viral diarrhea virus isolated from dairy cattle in Northwestern China. *Virus Genes* **2013**, *46*, 375-376, doi:10.1007/s11262-012-0861-3.
89. Gonzalez Altamiranda, E.A.; Kaiser, G.G.; Weber, N.; Leunda, M.R.; Pecora, A.; Malacari, D.A.; Moran, O.; Campero, C.M.; Odeon, A.C. Clinical and reproductive consequences of using BVDV-contaminated semen in artificial insemination in a beef herd in Argentina. *Anim Reprod Sci* **2012**, *133*, 146-152, doi:10.1016/j.anireprosci.2012.06.022.
90. Gonzalez-Bautista, E.D.D.; Bulla-Castaneda, D.M.; Lopez-Buitrago, H.A.; Diaz-Anaya, A.M.; Lancheros-Buitrago, D.J.; Garcia-Corredor, D.J.; Torreglosa, J.C.T.; Ortega, D.O.; Pulido-Medellin, M.O. Seroprevalence of bovine viral diarrhea virus (BVDV) in cattle from Sotaquira, Colombia. *Vet Anim Sci* **2021**, *14*, 100202, doi:10.1016/j.vas.2021.100202.
91. Graham, D.A.; Clegg, T.A.; O'Sullivan, P.; More, S.J. Influence of the retention of PI calves identified in 2012 during the voluntary phase of the Irish national bovine viral diarrhoea virus (BVDV) eradication programme on herd-level outcomes in 2013. *Prev Vet Med* **2015**, *120*, 298-305, doi:10.1016/j.prevetmed.2015.04.019.
92. Graham, D.A.; German, A.; McLaren, I.E.; Fitzpatrick, D.A. Testing of bulk tank milk from Northern Ireland dairy herds for viral RNA and antibody to bovine viral diarrhoea virus. *Vet Rec* **2001**, *149*, 261-265, doi:10.1136/vr.149.9.261.
93. Grooms, D.L.; Keilen, E.D. Screening of neonatal calves for persistent infection with bovine viral diarrhea virus by immunohistochemistry on skin biopsy samples. *Clin Diagn Lab Immunol* **2002**, *9*, 898-900, doi:10.1128/cdli.9.4.898-900.2002.
94. Guarino, H.; Nunez, A.; Repiso, M.V.; Gil, A.; Dargatz, D.A. Prevalence of serum antibodies to bovine herpesvirus-1 and bovine viral diarrhea virus in beef cattle in Uruguay. *Prev Vet Med* **2008**, *85*, 34-40, doi:10.1016/j.prevetmed.2007.12.012.

95. Guo, T.; Zhang, J.; Chen, X.; Wei, X.; Wu, C.; Cui, Q.; Hao, Y. Investigation of viral pathogens in cattle with bovine respiratory disease complex in Inner Mongolia, China. *Microb Pathog* **2021**, *153*, 104594, doi:10.1016/j.micpath.2020.104594.
96. Gupta, V.; Mishra, N.; Rajukumar, K.; Behera, S.P.; Dubey, P. Detection of bovine viral diarrhoea virus (BVDV) in peripheral blood mononuclear cells (PBMCs) of BVDV antibody positive Indian cattle following mitogen stimulation. *Indian J Anim Sci* **2014**, *84*, 156-158.
97. Gür, S. Prevalência de infecções por vírus da diarreia viral bovina e herpesvírus tipos 1 e 4 em fêmeas bovinas repetidoras do cio na região oeste da Turquia. *Brazilian Journal of Veterinary Research and Animal Science* **2011**, *48*, 228-233, doi:10.11606/s1413-95962011000300007.
98. Hagglund, S.; Svensson, C.; Emanuelson, U.; Valarcher, J.F.; Alenius, S. Dynamics of virus infections involved in the bovine respiratory disease complex in Swedish dairy herds. *Vet J* **2006**, *172*, 320-328, doi:10.1016/j.tvjl.2005.04.029.
99. Haines, D.M.; Martin, K.M.; Clark, E.G.; Jim, G.K.; Janzen, E.D. The immunohistochemical detection of Mycoplasma bovis and bovine viral diarrhea virus in tissues of feedlot cattle with chronic, unresponsive respiratory disease and/or arthritis. *Can Vet J* **2001**, *42*, 857-860.
100. Haines, D.M.; Moline, K.M.; Sargent, R.A.; Campbell, J.R.; Myers, D.J.; Doig, P.A. Immunohistochemical study of Hemophilus somnus, Mycoplasma bovis, Mannheimia hemolytica, and bovine viral diarrhea virus in death losses due to myocarditis in feedlot cattle. *Can Vet J* **2004**, *45*, 231-234.
101. Han, D.G.; Ryu, J.H.; Park, J.; Choi, K.S. Identification of a new bovine viral diarrhea virus subtype in the Republic of Korea. *Bmc Vet Res* **2018**, *14*, 233, doi:10.1186/s12917-018-1555-4.
102. Handel, I.G.; Willoughby, K.; Land, F.; Koterwas, B.; Morgan, K.L.; Tanya, V.N.; Bronsvoort, B.M. Seroepidemiology of bovine viral diarrhoea virus (BVDV) in the Adamawa Region of Cameroon and use of the SPOT test to identify herds with PI calves. *PLoS One* **2011**, *6*, e21620, doi:10.1371/journal.pone.0021620.
103. Hanon, J.B.; De Baere, M.; de la Ferte, C.; Roelandt, S.; Guillot, G.; Van der Stede, Y.; Cay, B. Serological monitoring on milk and serum samples in a BVD eradication program: A field study in Belgium showing antibody ELISA performances and epidemiological aspects. *Prev Vet Med* **2018**, *160*, 136-144, doi:10.1016/j.prevetmed.2018.07.008.
104. Hanon, J.B.; Van der Stede, Y.; Antonissen, A.; Mullender, C.; Tignon, M.; van den Berg, T.; Caij, B. Distinction between persistent and transient infection in a bovine viral diarrhoea (BVD) control programme: appropriate interpretation of real-time RT-PCR and antigen-ELISA test results. *Transbound Emerg Dis* **2014**, *61*, 156-162, doi:10.1111/tbed.12011.
105. Hashemi, M.; Bakhshesh, M.; Khezri, M.; Gharagouzlouian, M.M.; Tavakoli, G. A two-year serological study of bovine viral diarrhea virus, bovine alphaherpesvirus 1 and bovine parainfluenza virus type 3 in Qazvin dairy cattle farms, Northwestern of Iran. *Veterinarski arhiv* **2022**, *92*, 1-10, doi:10.24099/vet.arhiv.1123.
106. Hashemi, M.; Bakhshesh, M.; Manavian, M. Bovine Viral Diarrhea Virus and Bovine Herpes Virus-1 in Dairy Cattle Herds in Fars Province, Southern Iran: Seroprevalence and Evaluation of Risk Factors. *Arch Razi Inst* **2022**, *77*, 1621-1629, doi:10.22092/ARI.2022.356904.1941.
107. Hay, K.E.; Ambrose, R.C.; Morton, J.M.; Horwood, P.F.; Gravel, J.L.; Waldron, S.; Commins, M.A.; Fowler, E.V.; Clements, A.C.; Barnes, T.S.; et al. Effects of exposure to Bovine viral diarrhoea virus 1 on risk of bovine respiratory disease in Australian feedlot cattle. *Prev Vet Med* **2016**, *126*, 159-169, doi:10.1016/j.prevetmed.2016.01.025.
108. Hay, K.E.; Barnes, T.S.; Morton, J.M.; Gravel, J.L.; Commins, M.A.; Horwood, P.F.; Ambrose, R.C.; Clements, A.C.; Mahony, T.J. Associations between exposure to viruses and bovine respiratory disease in Australian feedlot cattle. *Prev Vet Med* **2016**, *127*, 121-133, doi:10.1016/j.prevetmed.2016.01.024.
109. Helal, M.A.; Okamatsu, H.; Tajima, M. Bovine viral diarrhea virus infection in a dairy herd with high prevalence of persistently infected calves. *Jpn J Vet Res* **2012**, *60*, 111-117.

110. Hessman, B.E.; Fulton, R.W.; Sjeklocha, D.B.; Murphy, T.A.; Ridpath, J.F.; Payton, M.E. Evaluation of economic effects and the health and performance of the general cattle population after exposure to cattle persistently infected with bovine viral diarrhea virus in a starter feedlot. *Am J Vet Res* **2009**, *70*, 73-85, doi:10.2460/ajvr.70.1.73.
111. Hilbe, M.; Stalder, H.; Peterhans, E.; Haessig, M.; Nussbaumer, M.; Egli, C.; Schelp, C.; Zlinszky, K.; Ehrensperger, F. Comparison of five diagnostic methods for detecting bovine viral diarrhea virus infection in calves. *J Vet Diagn Invest* **2007**, *19*, 28-34, doi:10.1177/104063870701900105.
112. Hill, F.I.; Reichel, M.P.; McCoy, R.J.; Tisdall, D.J. Evaluation of two commercial enzyme-linked immunosorbent assays for detection of bovine viral diarrhoea virus in serum and skin biopsies of cattle. *N Z Vet J* **2007**, *55*, 45-48, doi:10.1080/00480169.2007.36734.
113. Hoar, B.R.; McQuarry, A.C.; Hietala, S.K. Prevalence of Neospora caninum and persistent infection with bovine viral diarrhea virus in dairy-breed steers in a feedlot. *J Am Vet Med Assoc* **2007**, *230*, 1038-1043, doi:10.2460/javma.230.7.1038.
114. Holroyd, R.; Taylor, J.; McGowan, M. Impact of infectious diseases on beef cattle reproduction. **2012**.
115. Hoppe, I.; Medeiros, A.S.R.; Arns, C.W.; Samara, S.I. Bovine respiratory syncytial virus seroprevalence and risk factors in non-vaccinated dairy cattle herds in Brazil. *Bmc Vet Res* **2018**, *14*, 208, doi:10.1186/s12917-018-1535-8.
116. Hoppe, I.; Souza-Pollo, A.; Medeiros, A.S.R.; Samara, S.I.; Carvalho, A.A.B. HoBi-like pestivirus infection in an outbreak of bovine respiratory disease. *Res Vet Sci* **2019**, *126*, 184-191, doi:10.1016/j.rvsc.2019.09.003.
117. Hotchkiss, E.J.; Dagleish, M.P.; Willoughby, K.; McKendrick, I.J.; Finlayson, J.; Zadoks, R.N.; Newsome, E.; Brulisauer, F.; Gunn, G.J.; Hodgson, J.C. Prevalence of Pasteurella multocida and other respiratory pathogens in the nasal tract of Scottish calves. *Vet Rec* **2010**, *167*, 555-560, doi:10.1136/vr.c4827.
118. Hou, P.; Zhao, G.; Wang, H.; He, H. Prevalence of bovine viral diarrhea virus in dairy cattle herds in eastern China. *Trop Anim Health Prod* **2019**, *51*, 791-798, doi:10.1007/s11250-018-1751-z.
119. Ince, O.B.; Sevik, M. Risk assessment and seroprevalence of bovine herpesvirus type 1 infection in dairy herds in the inner Aegean Region of Turkey. *Comp Immunol Microbiol Infect Dis* **2022**, *80*, 101741, doi:10.1016/j.cimid.2021.101741.
120. Ince, O.B.; Sevik, M.; Ozgur, E.G.; Sait, A. Risk factors and genetic characterization of bovine respiratory syncytial virus in the inner Aegean Region, Turkey. *Trop Anim Health Prod* **2021**, *54*, 4, doi:10.1007/s11250-021-03022-5.
121. Jawor, P.; Krol, D.; Mee, J.F.; Soltysiak, Z.; Dzimira, S.; Larska, M.; Stefaniak, T. Infection exposure, detection and causes of death in perinatal mortalities in Polish dairy herds. *Theriogenology* **2017**, *103*, 130-136, doi:10.1016/j.theriogenology.2017.07.044.
122. Jeoung, H.Y.; Lim, J.A.; Jeong, W.; Oem, J.K.; An, D.J. Three clusters of bovine kobuvirus isolated in Korea, 2008-2010. *Virus Genes* **2011**, *42*, 402-406, doi:10.1007/s11262-011-0593-9.
123. Kabongo, N.; Van Vuuren, M. Detection of bovine viral diarrhoea virus in specimens from cattle in South Africa and possible association with clinical disease. *J S Afr Vet Assoc* **2004**, *75*, 90-93, doi:10.4102/jsava.v75i2.459.
124. Kadohira, M.; Tajima, M. A case control study of Bovine Viral Diarrhea Virus (BVDV) Persistent Infection (PI) in Betsukai, Hokkaido, Japan. *J Vet Med Sci* **2010**, *72*, 635-638, doi:10.1292/jvms.09-0305.
125. Kaiser, V.; Nebel, L.; Schupbach-Regula, G.; Zanoni, R.G.; Schweizer, M. Influence of border disease virus (BDV) on serological surveillance within the bovine virus diarrhea (BVD) eradication program in Switzerland. *Bmc Vet Res* **2017**, *13*, 21, doi:10.1186/s12917-016-0932-0.

126. Kale, M.; Ozturk, D.; Hasircioglu, S.; Pehlivanoglu, F.; Turutoglu, H. Some viral and bacterial respiratory tract infections of dairy cattle during the summer season. *Acta Vet.* **2013**, *63*, 227-236, doi:10.2298/avb1303227k.
127. Kale, M.; Yavru, S.; Ata, A.; Kocamuftuoglu, M.; Yaplcl, O.; Haslrcloglu, S. Bovine viral diarrhea virus (BVDV) infection in relation to fertility in heifers. *J Vet Med Sci* **2011**, *73*, 331-336, doi:10.1292/jvms.10-0254.
128. Kampa, J.; Alenius, S.; Emanuelson, U.; Chanlun, A.; Aiumlamai, S. Bovine herpesvirus type 1 (BHV-1) and bovine viral diarrhoea virus (BVDV) infections in dairy herds: self clearance and the detection of seroconversions against a new atypical pestivirus. *Vet J* **2009**, *182*, 223-230, doi:10.1016/j.tvjl.2008.07.006.
129. Kampa, J.; Stahl, K.; Moreno-Lopez, J.; Chanlun, A.; Aiumlamai, S.; Alenius, S. BVDV and BHV-1 infections in dairy herds in northern and northeastern Thailand. *Acta Vet Scand* **2004**, *45*, 181-192, doi:10.1186/1751-0147-45-181.
130. Kane, S.E.; Holler, L.D.; Braun, L.J.; Neill, J.D.; Young, D.B.; Ridpath, J.F.; Chase, C.C. Bovine viral diarrhea virus outbreak in a beef cow herd in South Dakota. *J Am Vet Med Assoc* **2015**, *246*, 1358-1362, doi:10.2460/javma.246.12.1358.
131. Karimi, O.; Bitaraf Sani, M.; Bakhshesh, M.; Harofteh, J.Z.; Poormirzayee, H. Seroprevalence of bovine viral diarrhea virus antibodies and risk factors in dairy cattle from the central desert of Iran. *Trop Anim Health Prod* **2022**, *54*, 176, doi:10.1007/s11250-022-03180-0.
132. Katoch, S.; Dohru, S.; Sharma, M.; Vashist, V.; Chahota, R.; Dhar, P.; Thakur, A.; Verma, S. Seroprevalence of viral and bacterial diseases among the bovines in Himachal Pradesh, India. *Vet World* **2017**, *10*, 1421-1426, doi:10.14202/vetworld.2017.1421-1426.
133. Khalili, M.; Molaei, M.M.; Mozaffari, A.A.; Giraei, F.D.; Ehsan, N.-f. Prevalence of bovine viral diarrhea virus antibodies in dairy cattle herds in the suburb of Kerman, Iran. *Comparative Clinical Pathology* **2011**, *21*, 1183-1185, doi:10.1007/s00580-011-1258-8.
134. Khodakaram-Tafti, A.; Mohammadi, A.; Farjani Kish, G.H. Molecular characterization and phylogenetic analysis of bovine viral diarrhea virus in dairy herds of Fars province, Iran. *Iran J Vet Res* **2016**, *17*, 89-97.
135. Kishimoto, M.; Tsuchiaka, S.; Rahpaya, S.S.; Hasebe, A.; Otsu, K.; Sugimura, S.; Kobayashi, S.; Komatsu, N.; Nagai, M.; Omatsu, T.; et al. Development of a one-run real-time PCR detection system for pathogens associated with bovine respiratory disease complex. *J Vet Med Sci* **2017**, *79*, 517-523, doi:10.1292/jvms.16-0489.
136. Klima, C.L.; Zaheer, R.; Cook, S.R.; Booker, C.W.; Hendrick, S.; Alexander, T.W.; McAllister, T.A. Pathogens of bovine respiratory disease in North American feedlots conferring multidrug resistance via integrative conjugative elements. *J Clin Microbiol* **2014**, *52*, 438-448, doi:10.1128/JCM.02485-13.
137. Kliučinskas, R.; Lukauskas, K.; Milius, J.; Vyšniauskis, G.; Kliučinskas, D.; Šalomskas, A. Detection of bovine viral diarrhoea virus in saliva samples. *Bulletin of the Veterinary Institute in Pulawy* **2008**, *52*, 31-37.
138. Konnai, S.; Mingala, C.N.; Sato, M.; Abes, N.S.; Venturina, F.A.; Gutierrez, C.A.; Sano, T.; Omata, Y.; Cruz, L.C.; Onuma, M.; et al. A survey of abortifacient infectious agents in livestock in Luzon, the Philippines, with emphasis on the situation in a cattle herd with abortion problems. *Acta Trop* **2008**, *105*, 269-273, doi:10.1016/j.actatropica.2007.12.004.
139. Kovago, C.; Forgach, P.; Szabara, A.; Mandoki, M.; Hornyak, A.; Duignan, C.; Pasztine Gere, E.; Rusvai, M. Seroprevalence of bovine viral diarrhoea virus in Hungary - situation before launching an eradication campaign. *Acta Vet Hung* **2015**, *63*, 255-263, doi:10.1556/AVet.2015.023.
140. Kozasa, T.; Tajima, M.; Yasutomi, I.; Sano, K.; Ohashi, K.; Onuma, M. Relationship of bovine viral diarrhea virus persistent infection to incidence of diseases on dairy farms based on bulk tank milk test by RT-PCR. *Vet Microbiol* **2005**, *106*, 41-47, doi:10.1016/j.vetmic.2004.12.009.

141. Kučer, N.; Marković, E.; Prpić, J.; Rudan, D.; Jemeršić, L.; Hajek, Z.; Škaro, K.; Pavlak, M.; Rudan, N. The epidemiology of bovine viral diarrhea virus infection on a dairy farm - clinical signs, seroprevalence, virus detection and genotyping. *Veterinarski arhiv* **2022**, *92*, 119-126, doi:10.24099/vet.arhiv.1028.
142. Kumar, S.K.; Palanivel, K.M.; Sukumar, K.; Ronald, B.S.M.; Selvaraju, G.; Ponnudurai, G. Herd-level risk factors for bovine viral diarrhea infection in cattle of Tamil Nadu. *Trop Anim Health Prod* **2018**, *50*, 793-799, doi:10.1007/s11250-017-1497-z.
143. Kuta, A.; Polak, M.P.; Larska, M.; Żmudziński, J.F. Monitoring of Bovine Viral Diarrhoea Virus (Bvdv) Infection in Polish Dairy Herds Using Bulk Tank Milk Samples. *Bulletin of the Veterinary Institute in Pulawy* **2013**, *57*, 149-156, doi:10.2478/bvip-2013-0028.
144. Larson, R.L.; Miller, R.B.; Kleiboeker, S.B.; Miller, M.A.; White, B.J. Economic costs associated with two testing strategies for screening feeder calves for persistent infection with bovine viral diarrhea virus. *J Am Vet Med Assoc* **2005**, *226*, 249-254, doi:10.2460/javma.2005.226.249.
145. Lassen, B.; Orro, T.; Aleksejev, A.; Raaperi, K.; Jarvis, T.; Viltrop, A. Neospora caninum in Estonian dairy herds in relation to herd size, reproduction parameters, bovine virus diarrhoea virus, and bovine herpes virus 1. *Vet Parasitol* **2012**, *190*, 43-50, doi:10.1016/j.vetpar.2012.05.021.
146. Lee, D.H.; Park, S.W.; Choi, E.W.; Lee, C.W. Investigation of the prevalence of bovine viral diarrhoea virus in dairy cows in South Korea. *Vet Rec* **2008**, *162*, 211-213, doi:10.1136/vr.162.7.211.
147. Lee, S.H.; Kim, H.Y.; Choi, E.W.; Kim, D. Causative agents and epidemiology of diarrhea in Korean native calves. *J Vet Sci* **2019**, *20*, e64, doi:10.4142/jvs.2019.20.e64.
148. Leon, J.C.P.; Diaz, W.; Vasquez, M.C.; Tobon, J.C.; Sanchez, A.; Ortiz, D. Seroprevalence and risk factor associated with respiratory viral pathogens in dual-purpose cattle of Aguachica, Rio de Oro, and La Gloria municipalities in Cesar department, Colombia. *Vet World* **2019**, *12*, 951-958, doi:10.14202/vetworld.2019.951-958.
149. Liang, H.; Geng, J.; Bai, S.; Aimuguri, A.; Gong, Z.; Feng, R.; Shen, X.; Wei, S. TaqMan real-time PCR for detecting bovine viral diarrhea virus. *Pol J Vet Sci* **2019**, *22*, 405-413, doi:10.24425/pjvs.2019.129300.
150. Liebler-Tenorio, E.M.; Kenklies, S.; Greiser-Wilke, I.; Makoschey, B.; Pohlenz, J.F. Incidence of BVDV1 and BVDV2 infections in cattle submitted for necropsy in Northern Germany. *J Vet Med B Infect Dis Vet Public Health* **2006**, *53*, 363-369, doi:10.1111/j.1439-0450.2006.00992.x.
151. Loneragan, G.H.; Gould, D.H.; Mason, G.L.; Garry, F.B.; Yost, G.S.; Miles, D.G.; Hoffman, B.W.; Mills, L.J. Involvement of microbial respiratory pathogens in acute interstitial pneumonia in feedlot cattle. *Am J Vet Res* **2001**, *62*, 1519-1524, doi:10.2460/ajvr.2001.62.1519.
152. Loneragan, G.H.; Thomson, D.U.; Montgomery, D.L.; Mason, G.L.; Larson, R.L. Prevalence, outcome, and health consequences associated with persistent infection with bovine viral diarrhea virus in feedlot cattle. *J Am Vet Med Assoc* **2005**, *226*, 595-601, doi:10.2460/javma.2005.226.595.
153. Lucchese, L.; Benkirane, A.; Hakimi, I.; El Idrissi, A.; Natale, A. Seroprevalence study of the main causes of abortion in dairy cattle in Morocco. *Vet Ital* **2016**, *52*, 13-19, doi:10.12834/VetIt.388.1813.1.
154. Luzzago, C.; Frigerio, M.; Tolari, F.; Mazzei, M.; Salvadori, C.; Del Piero, F.; Arispici, M. Indirect immunohistochemistry on skin biopsy for the detection of persistently infected cattle with bovine viral diarrhoea virus in Italian dairy herds. *New Microbiol* **2006**, *29*, 127-131.
155. Lysholm, S.; Ramabu, S.S.; Berg, M.; Wensman, J.J. First-time detection of bovine viral diarrhoea virus, BVDV-1, in cattle in Botswana. *Onderstepoort J Vet Res* **2019**, *86*, e1-e7, doi:10.4102/ojvr.v86i1.1764.

156. Machado, G.; Egocheaga, R.M.; Hein, H.E.; Miranda, I.C.; Neto, W.S.; Almeida, L.L.; Canal, C.W.; Stein, M.C.; Corbellini, L.G. Bovine Viral Diarrhoea Virus (BVDV) in Dairy Cattle: A Matched Case-Control Study. *Transbound Emerg Dis* **2016**, *63*, e1-13, doi:10.1111/tbed.12219.
157. Mahmoud, M.A.; Allam, A.M. Seroprevalence of bovine viral diarrhea virus (BVDV), bovine herpes virus type 1 (BHV-1), parainfluenza type 3 virus (PI-3V) and bovine respiratory syncytial virus (BRSV) among non vaccinated cattle. *Global Vet.* **2013**, *10*, 348-353, doi:10.5829/idosi.gv.2013.10.3.72119.
158. Mainar-Jaime, R.C.; Berzal-Herranz, B.; Arias, P.; Rojo-Vazquez, F.A. Epidemiological pattern and risk factors associated with bovine viral-diarrhoea virus (BVDV) infection in a non-vaccinated dairy-cattle population from the Asturias region of Spain. *Prev Vet Med* **2001**, *52*, 63-73, doi:10.1016/s0167-5877(01)00239-2.
159. Marques, A.L.A.; Assis, A.C.d.O.; Simões, S.V.D.; Tolentino, M.L.D.d.L.; Azevedo, S.S.d. Risk factors associated with Bovine Viral Diarrhea Virus (BVDV) infection in the semiarid of the state of Paraíba, in the northeast region of Brazil. *Semina: Ciências Agrárias* **2016**, *37*, 3095-3105, doi:10.5433/1679-0359.2016v37n5p3095.
160. Martin, C.C.; Baccili, C.C.; Silva, B.T.; Novo, S.M.F.; Sobreira, N.M.; Pituco, E.M.; Gomes, V. Detection of Bovine Viral Diarrhea virus infection in newborn calves before colostrum intake. *Semina: Ciências Agrárias* **2016**, *37*, 1379-1388, doi:10.5433/1679-0359.2016v37n3p1379.
161. Martinez-Rodriguez, L.C.; Guzman-Barragan, B.L.; Ordonez, D.; Tafur-Gomez, G.A. Cattle seroprevalence and risk factors associated with bovine viral diarrhea in the northeastern of Colombia. *Trop Anim Health Prod* **2021**, *53*, 377, doi:10.1007/s11250-021-02774-4.
162. Maya, L.; Macias-Rioseco, M.; Silveira, C.; Giannitti, F.; Castells, M.; Salvo, M.; Rivero, R.; Cristina, J.; Gianneechini, E.; Puentes, R.; et al. An extensive field study reveals the circulation of new genetic variants of subtype 1a of bovine viral diarrhea virus in Uruguay. *Arch Virol* **2020**, *165*, 145-156, doi:10.1007/s00705-019-04446-z.
163. Maya, L.; Puentes, R.; Reolon, E.; Acuna, P.; Riet, F.; Rivero, R.; Cristina, J.; Colina, R. Molecular diversity of bovine viral diarrhea virus in uruguay. *Arch Virol* **2016**, *161*, 529-535, doi:10.1007/s00705-015-2688-4.
164. McCarthy, M.C.; O'Grady, L.; McAloon, C.G.; Mee, J.F. Longitudinal Prevalence of Antibodies to Endemic Pathogens in Bulk Tank Milk Samples From Dairy Herds Engaged or Not in Contract Heifer Rearing. *Front Vet Sci* **2021**, *8*, 785128, doi:10.3389/fvets.2021.785128.
165. McDougall, S. Effect of calf age on bovine viral diarrhea virus tests. *J Vet Diagn Invest* **2021**, *33*, 528-537, doi:10.1177/1040638721998821.
166. Mehmet, K.; A, A.; S, Y.; O, Y.; O, B.; M.S, G. The effect of infection with bovine viral diarrhea virus on the fertility of cows and heifers. *Acta Vet.* **2006**, *56*, 467-477, doi:10.2298/avb0606467k.
167. Meiring, T.; Prozesky, L.; Du Preez, E.R.; Verwoerd, D.J. The diagnosis and prevalence of persistent infection with bovine viral diarrhoea virus in South African feedlot cattle. *Onderstepoort J Vet Res* **2011**, *78*, 323, doi:10.4102/ojvr.v78i1.323.
168. Minami, F.; Nagai, M.; Ito, M.; Matsuda, T.; Takai, H.; Jinkawa, Y.; Shimano, T.; Hayashi, M.; Seki, Y.; Sakoda, Y.; et al. Reactivity and prevalence of neutralizing antibodies against Japanese strains of bovine viral diarrhea virus subgenotypes. *Comp Immunol Microbiol Infect Dis* **2011**, *34*, 35-39, doi:10.1016/j.cimid.2009.10.007.
169. Mineo, T.W.; Alenius, S.; Naslund, K.; Montassier, H.J.; Bjorkman, C. Distribution of antibodies against Neospora caninum, BVDV and BHV-1 among cows in brazilian dairy herds with reproductive disorders. *Brazil. J. Vet. Parasitol.* **2006**, *15*, 188-192.
170. Mockeliuniene, V.; Salomskas, A.; Mockeliunas, R.; Petkevicius, S. Prevalence and epidemiological features of bovine viral diarrhoea virus infection in Lithuania. *Vet Microbiol* **2004**, *99*, 51-57, doi:10.1016/j.vetmic.2003.11.008.
171. Moore, S.J.; O'Dea, M.A.; Perkins, N.; O'Hara, A.J. Estimation of nasal shedding and seroprevalence of organisms known to be associated with bovine respiratory disease in

Australian live export cattle. *J Vet Diagn Invest* **2015**, *27*, 6-17, doi:10.1177/1040638714559741.
172. Morton, J.M.; Phillips, N.J.; Taylor, L.F.; McGowan, M.R. Bovine viral diarrhoea virus in beef heifers in commercial herds in Australia: mob-level seroprevalences and incidences of seroconversion, and vaccine efficacy. *Aust Vet J* **2013**, *91*, 517-524, doi:10.1111/avj.12129.
173. Mõtus, R.V.O.V. Seroprevalence of selected endemic infectious diseases in large-scale Estonian dairy herds and their associations with cow longevity and culling rates. *Preventive veterinary medicine* **2021**.
174. Muasya, D.; Leeuwen, J.V.; Gitau, G.; McKenna, S.; Heider, L.; Muraya, J. Evaluation of antibody and antigen cross-reaction in Kenyan dairy cattle naturally infected with two pestiviruses: Bovine viral diarrhea virus and classical swine fever virus. *Vet World* **2022**, *15*, 1290-1296, doi:10.14202/vetworld.2022.1290-1296.
175. Munoz-Zanzi, C.A.; Hietala, S.K.; Thurmond, M.C.; Johnson, W.O. Quantification, risk factors, and health impact of natural congenital infection with bovine viral diarrhea virus in dairy calves. *Am J Vet Res* **2003**, *64*, 358-365, doi:10.2460/ajvr.2003.64.358.
176. Nikbakht, G.; Tabatabaei, S.; Lotfollahzadeh, S.; Nayeri Fasaei, B.; Bahonar, A.; Khormali, M. Seroprevalence of bovine viral diarrhoea virus, bovine herpesvirus 1 and bovine leukaemia virus in Iranian cattle and associations among studied agents. *J Appl Anim Res* **2014**, *43*, 22-25, doi:10.1080/09712119.2014.883995.
177. Nilnont, T.; Aiumlamai, S.; Kanistanont, K.; Inchaisri, C.; Kampa, J. Bovine viral diarrhea virus (BVDV) infection in dairy cattle herds in northeast Thailand. *Trop Anim Health Prod* **2016**, *48*, 1201-1208, doi:10.1007/s11250-016-1075-9.
178. Noaman, V.; Nabinejad, A.R. Seroprevalence and risk factors assessment of the three main infectious agents associated with abortion in dairy cattle in Isfahan province, Iran. *Trop Anim Health Prod* **2020**, *52*, 2001-2009, doi:10.1007/s11250-020-02207-8.
179. Nugroho, W.; Reichel, M.P.; Ruff, N.; Gazali, A.M.; Sakke, I.S. Infection with Bovine Viral Diarrhea Virus in Cattle in Southern Papua, Indonesia. *Acta Trop* **2020**, *212*, 105712, doi:10.1016/j.actatropica.2020.105712.
180. O'Connor, A.M.; Reed, M.C.; Denagamage, T.N.; Yoon, K.J.; Sorden, S.D.; Cooper, V.L. Prevalence of calves persistently infected with bovine viral diarrhea virus in beef cow-calf herds enrolled in a voluntary screening project. *J Am Vet Med Assoc* **2007**, *230*, 1691-1696, doi:10.2460/javma.230.11.1691.
181. O'Connor, A.M.; Sorden, S.D.; Apley, M.D. Association between the existence of calves persistently infected with bovine viral diarrhea virus and commingling on pen morbidity in feedlot cattle. *Am J Vet Res* **2005**, *66*, 2130-2134, doi:10.2460/ajvr.2005.66.2130.
182. O'Neill, R.; Mooney, J.; Connaghan, E.; Furphy, C.; Graham, D.A. Patterns of detection of respiratory viruses in nasal swabs from calves in Ireland: a retrospective study. *Vet Rec* **2014**, *175*, 351, doi:10.1136/vr.102574.
183. Ochirkhuu, N.; Konnai, S.; Odbileg, R.; Odzaya, B.; Gansukh, S.; Murata, S.; Ohashi, K. Molecular detection and characterization of bovine viral diarrhea virus in Mongolian cattle and yaks. *Arch Virol* **2016**, *161*, 2279-2283, doi:10.1007/s00705-016-2890-z.
184. Oem, J.K.; Chung, J.Y.; Roh, I.S.; Kim, H.R.; Bae, Y.C.; Lee, K.H.; Jin, Y.H.; Lee, O.S. Characterization and phylogenetic analysis of Bovine viral diarrhea virus in brain tissues from nonambulatory (downer) cattle in Korea. *J Vet Diagn Invest* **2010**, *22*, 518-523, doi:10.1177/104063871002200404.
185. Oem, J.K.; Hyun, B.H.; Cha, S.H.; Lee, K.K.; Kim, S.H.; Kim, H.R.; Park, C.K.; Joo, Y.S. Phylogenetic analysis and characterization of Korean bovine viral diarrhea viruses. *Vet Microbiol* **2009**, *139*, 356-360, doi:10.1016/j.vetmic.2009.06.017.
186. Okumu, T.A.; John, N.M.; Wabacha, J.K.; Tsuma, V.; VanLeeuwen, J. Seroprevalence of antibodies for bovine viral diarrhoea virus, Brucella abortus and Neospora caninum, and their

roles in the incidence of abortion/foetal loss in dairy cattle herds in Nakuru District, Kenya. *Bmc Vet Res* **2019**, *15*, 95, doi:10.1186/s12917-019-1842-8.
187. Oliveira, T.E.S.; Pelaquim, I.F.; Flores, E.F.; Massi, R.P.; Valdiviezo, M.J.J.; Pretto-Giordano, L.G.; Alfieri, A.A.; Saut, J.P.E.; Headley, S.A. Mycoplasma bovis and viral agents associated with the development of bovine respiratory disease in adult dairy cows. *Transbound Emerg Dis* **2020**, *67 Suppl 2*, 82-93, doi:10.1111/tbed.13223.
188. Oliveira, T.E.S.; Scuisato, G.S.; Pelaquim, I.F.; Cunha, C.W.; Cunha, L.S.; Flores, E.F.; Pretto-Giordano, L.G.; Lisboa, J.A.N.; Alfieri, A.A.; Saut, J.P.E.; et al. The Participation of a Malignant Catarrhal Fever Virus and Mycoplasma bovis in the Development of Single and Mixed Infections in Beef and Dairy Cattle With Bovine Respiratory Disease. *Front Vet Sci* **2021**, *8*, 691448, doi:10.3389/fvets.2021.691448.
189. Oliveira, V.H.S.; Dall Agnol, A.M.; Fritzen, J.T.T.; Lorenzetti, E.; Alfieri, A.A.; Alfieri, A.F. Microbial diversity involved in the etiology of a bovine respiratory disease outbreak in a dairy calf rearing unit. *Comp Immunol Microbiol Infect Dis* **2020**, *71*, 101494, doi:10.1016/j.cimid.2020.101494.
190. Olmo, L.; Dye, M.T.; Reichel, M.P.; Young, J.R.; Nampanya, S.; Khounsy, S.; Thomson, P.C.; Windsor, P.A.; Bush, R.D. Investigation of infectious reproductive pathogens of large ruminants: Are neosporosis, brucellosis, leptospirosis and BVDV of relevance in Lao PDR? *Acta Trop* **2018**, *177*, 118-126, doi:10.1016/j.actatropica.2017.10.007.
191. Olmo, L.; Reichel, M.P.; Windsor, P.A.; Suon, S.; Wahl, L.C.; Thomson, P.C.; Bush, R.D. Are infectious reproductive pathogens of large ruminants a threat to improving food security? An investigation from Cambodia. *Trop Anim Health Prod* **2021**, *53*, 480, doi:10.1007/s11250-021-02897-8.
192. Olum, M.O.; Mungube, E.O.; Njanja, J.; Kidali, J.; Njenga, E.; Maichomo, M.; Tsuma, V.T.; Mugambi, J. Seroprevalence of canine neosporosis and bovine viral diarrhoea in dairy cattle in selected regions of Kenya. *Transbound Emerg Dis* **2020**, *67 Suppl 2*, 154-158, doi:10.1111/tbed.13429.
193. Ortega, D.O.; Sarmiento, R.A.M.; Torreglosa, J.C.T.; Rocha, J.F. Prevalence and risk factors of bovine viral diarrhea in Colombian cattle. *Vet World* **2020**, *13*, 1487-1494, doi:10.14202/vetworld.2020.1487-1494.
194. Oruc, E.; Saglam, Y.S.; Sozdutmaz, I.; Kapakin, K.A.T.; Temur, A.; Altun, S. The Investigation of Bovine Viral Diarrhoea Virus Antigens with Immunofluorescence and Immunohistochemical Methods in Bovine Abortions. *Pak Vet J* **2015**, *35*, 426-429.
195. Ozer, E.; Duman, R. Study of Bovine Viral Diarrhea Virus (BVDV) Infection in Dairy Cattle. *J Anim Vet Adv* **2011**, *10*, 1557-1560, doi:10.3923/javaa.2011.1557.1560.
196. Padalino, B.; Cirone, F.; Zappaterra, M.; Tullio, D.; Ficco, G.; Giustino, A.; Ndiana, L.A.; Pratelli, A. Factors Affecting the Development of Bovine Respiratory Disease: A Cross-Sectional Study in Beef Steers Shipped From France to Italy. *Front Vet Sci* **2021**, *8*, 627894, doi:10.3389/fvets.2021.627894.
197. Paetsch, C.; Fenton, K.; Perrett, T.; Janzen, E.; Clark, T.; Shearer, J.; Jelinski, M. Prospective case-control study of toe tip necrosis syndrome (TTNS) in western Canadian feedlot cattle. *Can Vet J* **2017**, *58*, 247-254.
198. Paller, T.; Hostnik, P.; Pogacnik, M.; Toplak, I. The Prevalence of Ten Pathogens Detected by a Real-Time Pcr Method in Nasal Swab Samples Collected from Live Cattle with Respiratory Disease. *Slov Vet Res* **2017**, *54*, 101-107.
199. Pardon, B.; Alliet, J.; Boone, R.; Roelandt, S.; Valgaeren, B.; Deprez, P. Prediction of respiratory disease and diarrhea in veal calves based on immunoglobulin levels and the serostatus for respiratory pathogens measured at arrival. *Prev Vet Med* **2015**, *120*, 169-176, doi:10.1016/j.prevetmed.2015.04.009.

200. Pardon, B.; De Bleecker, K.; Dewulf, J.; Callens, J.; Boyen, F.; Catry, B.; Deprez, P. Prevalence of respiratory pathogens in diseased, non-vaccinated, routinely medicated veal calves. *Vet Rec* **2011**, *169*, 278, doi:10.1136/vr.d4406.
201. Park, H.-J.; Kim, G.-D.; Lee, C.-H.; Kang, M.-H.; Min, K.-S. Analysis of bovine viral diarrhoea virus in Korea dairy farms. *Indian Journal of Animal Research* **2016**, *50*, 224-229, doi:10.18805/ijar.9374.
202. Peddireddi, L.; Foster, K.A.; Poulsen, E.G.; An, B.; Hoang, Q.H.; O'Connell, C.; Anderson, J.W.; Thomson, D.U.; Hanzlicek, G.A.; Bai, J.; et al. Molecular detection and characterization of transient bovine viral diarrhea virus (BVDV) infections in cattle commingled with ten BVDV persistently infected cattle. *J Vet Diagn Invest* **2018**, *30*, 413-422, doi:10.1177/1040638717753962.
203. Pillars, R.B.; Grooms, D.L. Serologic evaluation of five unvaccinated heifers to detect herds that have cattle persistently infected with bovine viral diarrhea virus. *Am J Vet Res* **2002**, *63*, 499-505, doi:10.2460/ajvr.2002.63.499.
204. Quintero Barbosa, J.; Corredor Figueroa, A.P.; Salas, S.S.; Camargo, H.; Sanchez, A.; Tobon, J.; Ortiz, D.; Schachtebeck, E.; Gutierrez, M.F. High prevalence of persistently infected animals from bovine viral diarrhea in Colombian cattle. *Bmc Vet Res* **2019**, *15*, 23, doi:10.1186/s12917-018-1769-5.
205. Quintero Rodriguez, L.E.; Dominguez, G.; Alvarado Pinedo, M.F.; Traveria, G.E.; More, G.; Campero, L.M.; de la Sota, R.L.; Madoz, L.V.; Giuliodori, M.J. Association of bovine viral diarrhea virus, bovine herpesvirus 1, and Neospora caninum with late embryonic losses in highly supplemented grazing dairy cows. *Theriogenology* **2022**, *194*, 126-132, doi:10.1016/j.theriogenology.2022.10.002.
206. Qurat ul, A.; Ahmad, A.; Awan, F.N.; Rabbani, M.; Mushtaq, M.H. Molecular characterization and phylogeny of bovine viral diarrhea virus isolated from persistently infected exotic cattle in Punjab, Pakistan. *Pakistan Journal of Agricultural Sciences* **2022**, *59*, 677-683, doi:10.21162/Pakjas/22.478.
207. Raheem, A.; Ahmad, A.; Rabbani, M.; Ghafoor, A.; Ajnum, A.A.; Avais, M.; Qurat-ul-Ain; Ramizland, R.M.; Ur-Rehman, H. Determination of Sero-Prevalence and Associated Risk Factors of Bovine Viral Diarrhea Virus (Bvdv) in Bovine Population from Southern Punjab, Pakistan. *The Journal of Animal and Plant Sciences* **2020**, *30*, 545-551, doi:10.36899/japs.2020.3.0064.
208. Raizman, E.A.; Pogranichniy, R.; Negron, M.; Schnur, M.; Tobar-Lopez, D.E. Seroprevalence of infectious bovine rhinotracheitis and bovine viral diarrhea virus type 1 and type 2 in non-vaccinated cattle herds in the Pacific Region of Central Costa Rica. *Trop Anim Health Prod* **2011**, *43*, 773-778, doi:10.1007/s11250-010-9762-4.
209. Ramírez Vásquez, N.F.; Villar Argaiz, D.; Fernández Silva, J.A.; Londoño Pino, J.; Chaparro Gutiérrez, J.J.; Olivera Ángel, M.E. Seroprevalence and risk factors of several bovine viral diseases in dairy farms of San Pedro de los Milagros, Antioquia, Colombia. *CES Medicina Veterinaria y Zootecnia* **2016**, *11*, 15-25, doi:10.21615/cesmvz.11.1.2.
210. Ranjbar, M.M.; Enayati, S.; Lotfi, M.; Yousefi, A.R.; Azimi, S.M.; Mousavi, S.R.; Yazdansetad, S.; Karimi, G. Simultaneous Detection of Bovine Rotavirus (BRV) and Bovine Viral Diarrhea (BVD) virus in Diarrheic Stool Samples: A Comparative Study of Molecular and Serological approaches. *Infection Epidemiology and Microbiology* **2021**, *7*, 17-28, doi:10.52547/iem.7.1.17.
211. Read, A.J.; Gestier, S.; Parrish, K.; Finlaison, D.S.; Gu, X.; O'Connor, T.W.; Kirkland, P.D. Prolonged Detection of Bovine Viral Diarrhoea Virus Infection in the Semen of Bulls. *Viruses* **2020**, *12*, doi:10.3390/v12060674.
212. Rêgo, M.J.P.; Batista Filho, A.F.B.; Oliveira, P.R.F.d.; Borges, J.D.M.; França, C.A.B.d.; Ribeiro, C.P.; Pituco, E.M.; Pinheiro Junior, J.W. Epidemiological analysis of infection by the bovine viral

diarrhea virus on family farms in Brazil. *Semina: Ciências Agrárias* **2016**, *37*, 4119-4130, doi:10.5433/1679-0359.2016v37n6p4119.
213. Rikula, U.; Nuotio, L.; Aaltonen, T.; Ruoho, O. Bovine viral diarrhoea virus control in Finland 1998-2004. *Prev Vet Med* **2005**, *72*, 139-142; discussion 215-139, doi:10.1016/j.prevetmed.2005.08.010.
214. Robesova, B.; Kovarcik, K.; Vilcek, S. Genotyping of bovine viral diarrhoea virus isolates from the Czech Republic. *Veterinární medicína* **2009**, *54*, 393-398, doi:10.17221/3053-vetmed.
215. Rola, J.G.; Larska, M.; Grzeszuk, M.; Bocian, L.; Kuta, A.; Polak, M.P.; Rola, J. Bulk tank milk somatic cell counts in dairy herds with different bovine viral diarrhoea virus status in Poland. *Prev Vet Med* **2014**, *116*, 183-187, doi:10.1016/j.prevetmed.2014.06.007.
216. Roshtkhari, F.; Mohammadi, G.; Mayameei, A. Serological evaluation of relationship between viral pathogens (BHV-1, BVDV, BRSV, PI-3V, and Adeno3) and dairy calf pneumonia by indirect ELISA. *Trop Anim Health Prod* **2012**, *44*, 1105-1110, doi:10.1007/s11250-011-0046-4.
217. Rufenacht, J.; Schaller, P.; Audige, L.; Knutti, B.; Kupfer, U.; Peterhans, E. The effect of infection with bovine viral diarrhea virus on the fertility of Swiss dairy cattle. *Theriogenology* **2001**, *56*, 199-210, doi:10.1016/s0093-691x(01)00556-8.
218. Rufenacht, J.; Schaller, P.; Audige, L.; Strasser, M.; Peterhans, E. Prevalence of cattle infected with bovine viral diarrhoea virus in Switzerland. *Vet Rec* **2000**, *147*, 413-417, doi:10.1136/vr.147.15.413.
219. Rush, D.M.; Thurmond, M.C.; Munoz-Zanzi, C.A.; Hietala, S.K. Descriptive epidemiology of postnatal bovine viral diarrhea virus infection in intensively managed dairy heifers. *J Am Vet Med Assoc* **2001**, *219*, 1426-1431, doi:10.2460/javma.2001.219.1426.
220. Rypula, K.; Ploneczka-Janeczko, K.; Czopowicz, M.; Klimowicz-Bodys, M.D.; Shabunin, S.; Siegwalt, G. Occurrence of BVDV Infection and the Presence of Potential Risk Factors in Dairy Cattle Herds in Poland. *Animals (Basel)* **2020**, *10*, doi:10.3390/ani10020230.
221. Ryu, J.H.; Choi, K.S. Genetic analysis of bovine viral diarrhea virus in pre-weaned native Korean calves. *Trop Anim Health Prod* **2019**, *51*, 2085-2090, doi:10.1007/s11250-019-01882-6.
222. S, Y.; A, S.; O, Y.; M, K. Serological evaluation of viral infections in bovine respiratory tract. *Acta Vet.* **2005**, *55*, 219-226, doi:10.2298/avb0503219y.
223. S. Sirag, M.; L. El Sayed, E.; M. Hussein, M.; A. El-Nesr, K.; B. El-Begawey, M. Immune-Pathological Studies on the Distribution and Localization of Bovine Viral Diarrhea Virus. *Advances in Animal and Veterinary Sciences* **2022**, *10*, 1916-1923, doi:10.17582/journal.aavs/2022/10.9.1916.1923.
224. Saa, L.R.; Perea, A.; Garcia-Bocanegra, I.; Arenas, A.J.; Jara, D.V.; Ramos, R.; Carbonero, A. Seroprevalence and risk factors associated with bovine viral diarrhea virus (BVDV) infection in non-vaccinated dairy and dual purpose cattle herds in Ecuador. *Trop Anim Health Prod* **2012**, *44*, 645-649, doi:10.1007/s11250-011-9948-4.
225. Sadam, D.H.; Alsaad, K.M. Bovine viral diarrhea and persistently infection of cattle at Nineveh province, Iraq. *Basrah J Vet Res* **2018**, *17*, 14-32.
226. Saeed, I.; Ali, Y.; Taha, K.; Mohammed, N.; Nouri, Y.; Mohammed, B.; Mohammed, O.; Elmagbool, S.; Elghazali, F. First report of Bovine Viral Diarrhea Virus antigen from pneumonic cattle in Sudan. *Journal of Advanced Veterinary and Animal Research* **2015**, *2*, 153-157, doi:10.5455/javar.2015.b67.
227. Saegerman, C.; Gaudino, M.; Savard, C.; Broes, A.; Ariel, O.; Meyer, G.; Ducatez, M.F. Influenza D virus in respiratory disease in Canadian, province of Quebec, cattle: Relative importance and evidence of new reassortment between different clades. *Transbound Emerg Dis* **2022**, *69*, 1227-1245, doi:10.1111/tbed.14085.
228. Safarpoor Dehkordi, F. Prevalence study of Bovine viral diarrhea virus by evaluation of antigen capture ELISA and RT-PCR assay in Bovine, Ovine, Caprine, Buffalo and Camel aborted fetuses in Iran. *AMB Express* **2011**, *1*, 32, doi:10.1186/2191-0855-1-32.

229. Sager, H.; Fischer, I.; Furrer, K.; Strasser, M.; Waldvogel, A.; Boerlin, P.; Audige, L.; Gottstein, B. A Swiss case-control study to assess Neospora caninum-associated bovine abortions by PCR, histopathology and serology. *Vet Parasitol* **2001**, *102*, 1-15, doi:10.1016/s0304-4017(01)00524-6.
230. Saipinta, D.; Panyamongkol, T.; Chuammitri, P.; Suriyasathaporn, W. Reduction in Mortality of Calves with Bovine Respiratory Disease in Detection with Influenza C and D Virus. *Animals (Basel)* **2022**, *12*, doi:10.3390/ani12233252.
231. Sarangi, L.N.; Tharani, N.; Polapally, S.; Rana, S.K.; Thodangala, N.; Bahekar, V.S.; Prasad, A.; Chandrasekhar Reddy, R.V.; Surendra, K.; Gonuguntla, H.N.; et al. Infectious bovine abortions: observations from an organized dairy herd. *Braz J Microbiol* **2021**, *52*, 439-448, doi:10.1007/s42770-020-00414-x.
232. Sarchet, J.J.; Pollreisz, J.P.; Bechtol, D.T.; Blanding, M.R.; Saltman, R.L.; Taube, P.C. Limitations of bacterial culture, viral PCR, and tulathromycin susceptibility from upper respiratory tract samples in predicting clinical outcome of tulathromycin control or treatment of bovine respiratory disease in high-risk feeder heifers. *PLoS One* **2022**, *17*, e0247213, doi:10.1371/journal.pone.0247213.
233. Sarikaya, B.; Azkur, A.K.; Gazyagci, S.; Aslan, M.E. Genetic Variability of Bovine Viral Diarrhea Virus in the 5 '-UTR in the Central Anatolia of Turkey. *Acta Scientiae Veterinariae* **2012**, *40*.
234. Sarrazin, S.; Veldhuis, A.; Meroc, E.; Vangeel, I.; Laureyns, J.; Dewulf, J.; Caij, A.B.; Piepers, S.; Hooyberghs, J.; Ribbens, S.; et al. Serological and virological BVDV prevalence and risk factor analysis for herds to be BVDV seropositive in Belgian cattle herds. *Prev Vet Med* **2013**, *108*, 28-37, doi:10.1016/j.prevetmed.2012.07.005.
235. Sayers, R.G.; Byrne, N.; O'Doherty, E.; Arkins, S. Prevalence of exposure to bovine viral diarrhoea virus (BVDV) and bovine herpesvirus-1 (BoHV-1) in Irish dairy herds. *Res Vet Sci* **2015**, *100*, 21-30, doi:10.1016/j.rvsc.2015.02.011.
236. Schefers, J.; Munoz-Zanzi, C.; Collins, J.E.; Goyal, S.M.; Ames, T.R. Serological evaluation of precolostral serum samples to detect Bovine viral diarrhea virus infections in large commercial dairy herds. *J Vet Diagn Invest* **2008**, *20*, 625-628, doi:10.1177/104063870802000515.
237. Scott, H.M.; Sorensen, O.; Wu, J.T.Y.; Chow, E.Y.W.; Manninen, K.; VanLeeuwen, J.A. Seroprevalence of Mycobacterium avium subspecies paratuberculosis, Neospora caninum, Bovine leukemia virus, and Bovine viral diarrhea virus infection among dairy cattle and herds in Alberta and agroecological risk factors associated with seropositivity. *Canadian Veterinary Journal-Revue Veterinaire Canadienne* **2006**, *47*, 981-991.
238. Segura-Correa, J.C.; Zapata-Campos, C.C.; Jasso-Obregon, J.O.; Martinez-Burnes, J.; Lopez-Zavala, R. Seroprevalence and risk factors associated with bovine herpesvirus 1 and bovine viral diarrhea virus in North-Eastern Mexico. *Open Vet J* **2016**, *6*, 143-149, doi:10.4314/ovj.v6i2.12.
239. Seki, Y.; Seimiya, Y.M.; Motokawa, M.; Miyazaki, H.; Konno, M.; Yaegashi, G. Field application of the combination of neutralizing test and virus isolation, so-called spot test, to detect herds including cattle persistently infected with bovine viral diarrhea virus. *J Vet Med Sci* **2007**, *69*, 1087-1089, doi:10.1292/jvms.69.1087.
240. Seki, Y.; Seimiya, Y.M.; Yaegashi, G.; Sato, C. Identification of herds with cattle persistently infected with bovine viral diarrhea virus by virological evaluation of three calves. *J Vet Med Sci* **2006**, *68*, 255-258, doi:10.1292/jvms.68.255.
241. Selim, A.M.; Elhaig, M.M.; Moawed, S.A.; El-Nahas, E. Modeling the potential risk factors of bovine viral diarrhea prevalence in Egypt using univariable and multivariable logistic regression analyses. *Vet World* **2018**, *11*, 259-267, doi:10.14202/vetworld.2018.259-267.
242. Semra, O.-G.; Z, Y.; H, A.; D, C. Seroprevalence of bovine viral respiratory diseases. *Acta Vet.* **2007**, *57*, 11-16, doi:10.2298/avb0701011o.

243. Seyyal, A.K.; Firat, Y.; Hakan Bozkurt, H.; Gülyaz, V.; Kemal, A.K. The prevalence of bovine viral diarrhoea virus (BVDV) infections in cattle and existence of persistently infected cattle in the Trakya region. *Turkish Journal of Veterinary and Animal Sciences* **2002**, *26*, 245-248.
244. Shahriar, F.M.; Clark, E.G.; Janzen, E.; West, K.; Wobeser, G. Coinfection with bovine viral diarrhea virus and Mycoplasma bovis in feedlot cattle with chronic pneumonia. *Can Vet J* **2002**, *43*, 863-868.
245. Shirvani, E.; Lotfi, M.; Kamalzadeh, M.; Noaman, V.; Bahriari, M.; Morovati, H.; Hatami, A. Seroepidemiological study of bovine respiratory viruses (BRSV, BoHV-1, PI-3V, BVDV, and BAV-3) in dairy cattle in central region of Iran (Esfahan province). *Trop Anim Health Prod* **2012**, *44*, 191-195, doi:10.1007/s11250-011-9908-z.
246. Siegwart, N.; Hilbe, M.; Hassig, M.; Braun, U. Increased risk of BVDV infection of calves from pregnant dams on communal Alpine pastures in Switzerland. *Vet J* **2006**, *172*, 386-388, doi:10.1016/j.tvjl.2005.07.018.
247. Silva, B.P.e.; Soares, L.B.F.; Macêdo, A.A.d.; Oliveira, J.M.B.d.; Aragão, B.B.; Nascimento, S.A.d.; Pinheiro Júnior, J.W. Soroprevalência e fatores de risco associados ao herpesvírus bovino tipo 1 e ocorrência da infecção pelo vírus da diarreia viral bovina em vacas leiteiras no estado de Pernambuco. *Medicina Veterinária (UFRPE)* **2019**, *13*, 399-405, doi:10.26605/medvet-v13n3-3302.
248. Socha, W.; Larska, M.; Rola, J.; Bednarek, D. Occurrence of Bovine Coronavirus and other Major Respiratory Viruses in Cattle in Poland. *J Vet Res* **2022**, *66*, 479-486, doi:10.2478/jvetres-2022-0059.
249. Solis-Calderon, J.J.; Segura-Correa, V.M.; Segura-Correa, J.C. Bovine viral diarrhoea virus in beef cattle herds of Yucatan, Mexico: seroprevalence and risk factors. *Prev Vet Med* **2005**, *72*, 253-262, doi:10.1016/j.prevetmed.2005.06.004.
250. Song, M.C.; Choi, K.S. Phylogenetic analysis of bovine viral diarrhea virus from Korean indigenous calves in gyeongbuk province. *Journal of Veterinary Clinics* **2010**, *27*, 635-639.
251. Song, M.C.; Choi, K.S. Genetic characterization of bovine viral diarrhea virus from korean indigenous calves in gyeongbuk province. *Journal of Veterinary Clinics* **2010**, *27*, 220-224.
252. Sood, R.; Bhatia, S.; Gounalan, S.; Patil, S.S.; Pattnaik, B. Sero-prevalence of bovine viral diarrhoea virus in India: A survey from 1999-2004. *Indian J Anim Sci* **2007**, *77*, 227-229.
253. Souza, F.O.; Santos, G.A.d.; Souza, B.C.d.S.; Moreira, M.A.S.; Silva-Júnior, A.; Peixoto, R.d.M.; Costa, M.M.d. Frequency and risk factors associated with the bovine viral diarrhea virus in herds in the semiarid region of the states of Bahia and Pernambuco, Brazil. *Acta Veterinaria Brasilica* **2019**, *13*, 163-169, doi:10.21708/avb.2019.13.3.8554.
254. Spetter, M.J.; Louge Uriarte, E.L.; Armendano, J.I.; Alvarez, I.; Norero, N.S.; Storani, L.; Pereyra, S.B.; Verna, A.E.; Odeon, A.C.; Gonzalez Altamiranda, E.A. Frequency of bovine viral diarrhea virus (BVDV) in Argentinean bovine herds and comparison of diagnostic tests for BVDV detection in bovine serum samples: a preliminary study. *Braz J Microbiol* **2021**, *52*, 467-475, doi:10.1007/s42770-020-00410-1.
255. Stahl, K.; Rivera, H.; Vagsholm, I.; Moreno-Lopez, J. Bulk milk testing for antibody seroprevalences to BVDV and BHV-1 in a rural region of Peru. *Prev Vet Med* **2002**, *56*, 193-202, doi:10.1016/s0167-5877(02)00161-7.
256. Stephenson, M.K.; Palomares, R.A.; White, B.J.; Engelken, T.J.; Brock, K.V. Prevalence of bovine viral diarrhea virus (BVDV) persistently infected calves in auction markets from the southeastern United States; association between body weight and BVDV-positive diagnosis. *Prof. Build. Sci.* **2017**, *33*, 426-431, doi:10.15232/pas.2017-01619.
257. Sun, W.W.; Meng, Q.F.; Cong, W.; Shan, X.F.; Wang, C.F.; Qian, A.D. Herd-level prevalence and associated risk factors for Toxoplasma gondii, Neospora caninum, Chlamydia abortus and bovine viral diarrhoea virus in commercial dairy and beef cattle in eastern, northern and northeastern China. *Parasitol. Res.* **2015**, *114*, 4211-4218, doi:10.1007/s00436-015-4655-0.

258. Szabara, A.; Lang, Z.; Foldi, J.; Hornyak, A.; Abonyi, T.; Ozsvari, L. Prevalence of bovine viral diarrhoea virus in cattle farms in Hungary. *Acta Vet Hung* **2016**, *64*, 263-272, doi:10.1556/004.2016.026.
259. Szeredi, L.; Janosi, S.; Palfi, V. Microbiological and pathological examination of fatal calf pneumonia cases induced by bacterial and viral respiratory pathogens. *Acta Vet Hung* **2010**, *58*, 341-356, doi:10.1556/AVet.58.2010.3.7.
260. Talafha, A.Q.; Hirche, S.M.; Ababneh, M.M.; Al-Majali, A.M.; Ababneh, M.M. Prevalence and risk factors associated with bovine viral diarrhea virus infection in dairy herds in Jordan. *Trop Anim Health Prod* **2009**, *41*, 499-506, doi:10.1007/s11250-008-9214-6.
261. Talebkhan Garoussi, M.; Haghparast, A.; Hajenejad, M.R. Prevalence of Bovine Viral Diarrhoea Virus antibodies among the industrial dairy cattle herds in suburb of Mashhad-Iran. *Trop Anim Health Prod* **2009**, *41*, 663-667, doi:10.1007/s11250-008-9238-y.
262. Tamer, C.; Palanci, H.; Bayram, E.; Cakmaker, M.; Ozan, E.; Kadi, H.; Onkol, S.; Gumusova, S.; Albayrak, H. Serological data of bovine herpes virus type-1 and bovine viral diarrhea virus infections in various ruminants in small-scale farms in the Central and Eastern Black Sea Region, Turkey. *Indian Journal of Animal Research* **2017**, *52*, 903-906, doi:10.18805/ijar.v0iOF.8470.
263. Tavella, A.; Zambotto, P.; Stifter, E.; Lombardo, D.; Rabini, M.; Robatscher, E.; Brem, G. Investigation to the specificity of positive BVDV results in ear notch samples: Review on the five-year-old experience in the Autonomous Province of Bolzano (Italy). *Berl Munch Tierarztl* **2012**, *125*, 326-331, doi:10.2376/0005-9366-125-326.
264. Taylor, L.; Rodwell, B. Outbreak of foetal infection with bovine pestivirus in a central Queensland beef herd. *Aust Vet J* **2001**, *79*, 682-685, doi:10.1111/j.1751-0813.2001.tb10670.x.
265. Taylor, L.F.; Black, P.F.; Pitt, D.J.; Mackenzie, A.R.; Johnson, S.J.; Rodwell, B.J. A seroepidemiological study of bovine pestivirus in Queensland beef and dairy herds conducted in 1994/95. *Aust Vet J* **2006**, *84*, 163-168, doi:10.1111/j.1751-0813.2006.tb12771.x.
266. Tesfaye, A.; Omer, A.; Hussein, A.; Garoma, A.; Guyassa, C.; Paeshuyse, J.; Tolera, T.S. Seroprevalence of Bovine Viral Diarrhea Virus in Local Borana Cattle Breed and Camels (Camelus dromedarius) in Ethiopia. *Vet Med (Auckl)* **2021**, *12*, 141-148, doi:10.2147/VMRR.S305198.
267. Thabti, F.; Kassimi, L.B.; M'Zah, A.; Ben Romdane, S.; Russo, P.; Ben Said, M.S.; Hammami, S.; Pepin, M. First detection and genetic characterization of bovine viral diarrhoea viruses (BVDV) types 1 and 2 in Tunisia. *Revue De Medecine Veterinaire* **2005**, *156*, 419-422.
268. Thobokwe, G.; Heuer, C.; Hayes, D.P. Validation of a bulk tank milk antibody ELISA to detect dairy herds likely infected with bovine viral diarrhoea virus in New Zealand. *N Z Vet J* **2004**, *52*, 394-400, doi:10.1080/00480169.2004.36457.
269. Thompson, J.A.; de Miranda Henriques Leite, R.; Goncalves, V.S.; Leite, R.C.; Bandeira, D.A.; Herrmann, G.P.; Moreira, E.C.; Prado, P.E.; Lobato, Z.I.; de Brito, C.P.; et al. Spatial hierarchical variances and age covariances for seroprevalence to Leptospira interrogans serovar hardjo, BoHV-1 and BVDV for cattle in the State of Paraiba, Brazil. *Prev Vet Med* **2006**, *76*, 290-301, doi:10.1016/j.prevetmed.2006.05.010.
270. Timurkan, M.O.; Arslan, F.; Van Schalkwyk, A.; Pekmez, K.; Kaplan, M.; Cagirgan, A.A. Türkiye'nin Batısında Sığır Viral Diyare Virusunun (BVDV) Durumu: Üç Alt Grubun Tespiti. *Kafkas Universitesi Veteriner Fakultesi Dergisi* **2022**, *28*, 709-715, doi:10.9775/kvfd.2022.27881.
271. Toplak, I.; Hostnik, P.; Cerne, D.; Mrkun, J.; Staric, J. The Principles of the Voluntary Programme for the Control and Elimination of Bovine Viral Diarrhoea Virus (BVDV) From Infected Herds in Slovenia. *Front Vet Sci* **2021**, *8*, 676473, doi:10.3389/fvets.2021.676473.

272. Truyers, I.G.; Mellor, D.J.; Norquay, R.; Gunn, G.J.; Ellis, K.A. Eradication programme for bovine viral diarrhoea virus in Orkney 2001 to 2008. *Vet Rec* **2010**, *167*, 566-570, doi:10.1136/vr.c4944.
273. Tuncer, P.; Yesilbag, K. Serological detection of infection dynamics for respiratory viruses among dairy calves. *Vet Microbiol* **2015**, *180*, 180-185, doi:10.1016/j.vetmic.2015.08.025.
274. Tuncer-Goktuna, P.; Alpay, G.; Oner, E.B.; Yesilbag, K. The role of herpesviruses (BoHV-1 and BoHV-4) and pestiviruses (BVDV and BDV) in ruminant abortion cases in western Turkey. *Trop Anim Health Prod* **2016**, *48*, 1021-1027, doi:10.1007/s11250-016-1050-5.
275. Tutuncu, H.; Yazici, Z. Screening for Persistently Infected Cattle with Bovine Viral Diarrhea Virus in Small-Holder Cattle Farms Located in Samsun Province, Northern Turkey. *J Anim Plant Sci* **2016**, *26*, 291-293.
276. Uddin, M.A.; Ahasan, A.; Islam, K.; Islam, M.Z.; Mahmood, A.; Islam, A.; Islam, K.M.F.; Ahad, A. Seroprevalence of bovine viral diarrhea virus in crossbred dairy cattle in Bangladesh. *Vet World* **2017**, *10*, 906-913, doi:10.14202/vetworld.2017.906-913.
277. Ularamu, H.G.; Sibeko, K.P.; Bosman, A.B.; Venter, E.H.; van Vuuren, M. Genetic characterization of bovine viral diarrhoea (BVD) viruses: confirmation of the presence of BVD genotype 2 in Africa. *Arch Virol* **2013**, *158*, 155-163, doi:10.1007/s00705-012-1478-5.
278. VanLeeuwen, J.; Muraya, J.; Gitau, G.; Makau, D.; Crane, B.; McKenna, S.; Wichtel, J. Seroprevalence and risk factors of Neospora caninum and Bovine Viral Diarrhoea Virus in smallholder dairy cattle in Kenya. *East African Journal of Science, Technology and Innovation* **2021**, *3*, doi:10.37425/eajsti.v3i1.391.
279. VanLeeuwen, J.A.; Keefe, G.P.; Tremblay, R.; Power, C.; Wichtel, J.J. Seroprevalence of infection with Mycobacterium avium subspecies paratuberculosis, bovine leukemia virus, and bovine viral diarrhea virus in maritime Canada dairy cattle. *Can Vet J* **2001**, *42*, 193-198.
280. VanLeeuwen, J.A.; Tiwari, A.; Plaizier, J.C.; Whiting, T.L. Seroprevalences of antibodies against bovine leukemia virus, bovine viral diarrhea virus, Mycobacterium avium subspecies paratuberculosis, and Neospora caninum in beef and dairy cattle in Manitoba. *Can Vet J* **2006**, *47*, 783-786.
281. Varshney, P.; Qureshi, S.D.; Bhatia, A.K.; Pradhan, H.K.; Mishra, N.; Bhatia, S. Molecular and Antibody Based Diagnosis of Bovine Viral Diarrhea Virus (Bvdv) Associated with Cattle from Mathura, Uttar Pradesh (India). *Appl Biol Res* **2012**, *14*, 215-219.
282. Velasova, M.; Damaso, A.; Prakashbabu, B.C.; Gibbons, J.; Wheelhouse, N.; Longbottom, D.; Van Winden, S.; Green, M.; Guitian, J. Herd-level prevalence of selected endemic infectious diseases of dairy cows in Great Britain. *J Dairy Sci* **2017**, *100*, 9215-9233, doi:10.3168/jds.2016-11863.
283. Viana, R.B.; Kzam, A.d.S.L.; Monteiro, B.M.; Campello, C.C.; Sousa, E.D.M.; Souza, D.C.d.; Okuda, L.H.; Pituco, E.M.; Ribeiro Filho, J.D. Sensitivity and specificity of indirect ELISA for the detection of antibody titers against BVDV from beef cattle raised in Pará State. *Semina: Ciências Agrárias* **2017**, *38*, 3049-3058, doi:10.5433/1679-0359.2017v38n5p3049.
284. Vinomack, C.; Riviere, J.; Breard, E.; Viarouge, C.; Postic, L.; Zientara, S.; Vitour, D.; Belbis, G.; Spony, V.; Pagneux, C.; et al. Clinical cases of Bluetongue serotype 8 in calves in France in the 2018-2019 winter. *Transbound Emerg Dis* **2020**, *67*, 1401-1405, doi:10.1111/tbed.13466.
285. Waldner, C.; Wildman, B.K.; Hill, B.W.; Fenton, R.K.; Pittman, T.J.; Schunicht, O.C.; Jim, G.K.; Guichon, P.T.; Booker, C.W. Determination of the seroprevalence of Neospora caninum in feedlot steers in Alberta. *Can Vet J* **2004**, *45*, 218-224.
286. Waldner, C.L. Serological status for N. caninum, bovine viral diarrhea virus, and infectious bovine rhinotracheitis virus at pregnancy testing and reproductive performance in beef herds. *Anim Reprod Sci* **2005**, *90*, 219-242, doi:10.1016/j.anireprosci.2005.03.017.
287. Waldner, C.L.; Kennedy, R.I. Associations between health and productivity in cow-calf beef herds and persistent infection with bovine viral diarrhea virus, antibodies against bovine viral

diarrhea virus, or antibodies against infectious bovine rhinotracheitis virus in calves. *Am J Vet Res* **2008**, *69*, 916-927, doi:10.2460/ajvr.69.7.916.
288. Wang, H.; Wang, M.; Feng, X.; Li, Y.; Zhang, D.; Cheng, Y.; Liu, J.; Wang, X.; Zhang, L.; La, H.; et al. Genetic features of bovine viral diarrhea virus subgenotype 1c in newborn calves at nucleotide and synonymous codon usages. *Front Vet Sci* **2022**, *9*, 984962, doi:10.3389/fvets.2022.984962.
289. Wang, J.; Guo, Y.; Ning, X.; Liang, X.; Li, Y.; Kang, X.; Wang, X. Antibody detection and genotyping of bovine viral diarrhea virus in the dairy farms in Ningxia, China. *Journal of Biotech Research* **2022**, *12*, 192-198.
290. Weber, M.N.; Mosena, A.C.; Simoes, S.V.; Almeida, L.L.; Pessoa, C.R.; Budaszewski, R.F.; Silva, T.R.; Ridpath, J.F.; Riet-Correa, F.; Driemeier, D.; et al. Clinical Presentation Resembling Mucosal Disease Associated with 'HoBi'-like Pestivirus in a Field Outbreak. *Transbound Emerg Dis* **2016**, *63*, 92-100, doi:10.1111/tbed.12223.
291. Weber, M.N.; Silveira, S.; Machado, G.; Groff, F.H.; Mosena, A.C.; Budaszewski, R.F.; Dupont, P.M.; Corbellini, L.G.; Canal, C.W. High frequency of bovine viral diarrhea virus type 2 in Southern Brazil. *Virus Res* **2014**, *191*, 117-124, doi:10.1016/j.virusres.2014.07.035.
292. Weng, X.G.; Song, Q.J.; Wu, Q.; Liu, M.C.; Wang, M.L.; Wang, J.F. Genetic characterization of bovine viral diarrhea virus strains in Beijing, China and innate immune responses of peripheral blood mononuclear cells in persistently infected dairy cattle. *J Vet Sci* **2015**, *16*, 491-500, doi:10.4142/jvs.2015.16.4.491.
293. Wernicki, A.; Urban-Chmiel, R.; Stegierska, D.; Adaszek, L.; Kalinowski, M.; Puchalski, A.; Dec, M. Detection of the bovine viral diarrhoea virus (BVDV) in young beef cattle in eastern and south-eastern regions of Poland. *Pol J Vet Sci* **2015**, *18*, 141-146, doi:10.1515/pjvs-2015-0018.
294. Weston, J.F.; Heuer, C.; Parkinson, T.J.; Williamson, N.B. Causes of abortion on New Zealand dairy farms with a history of abortion associated with Neospora caninum. *N Z Vet J* **2012**, *60*, 27-34, doi:10.1080/00480169.2011.631171.
295. Williams, D.; Winden, S.V. Risk factors associated with high bulk milk antibody levels to common pathogens in UK dairies. *Vet Rec* **2014**, *174*, 580, doi:10.1136/vr.102049.
296. Wittum, T.E.; Grotelueschen, D.M.; Brock, K.V.; Kvasnicka, W.G.; Floyd, J.G.; Kelling, C.L.; Odde, K.G. Persistent bovine viral diarrhoea virus infection in US beef herds. *Prev Vet Med* **2001**, *49*, 83-94, doi:10.1016/s0167-5877(01)00181-7.
297. Yan, L.; Zhang, S.; Pace, L.; Wilson, F.; Wan, H.; Zhang, M. Combination of reverse transcription real-time polymerase chain reaction and antigen capture enzyme-linked immunosorbent assay for the detection of animals persistently infected with Bovine viral diarrhea virus. *J Vet Diagn Invest* **2011**, *23*, 16-25, doi:10.1177/104063871102300103.
298. Yesilbag, K.; Gungor, B. Seroprevalence of bovine respiratory viruses in North-Western Turkey. *Trop Anim Health Prod* **2008**, *40*, 55-60, doi:10.1007/s11250-007-9053-x.
299. Yilmaz, V. Prevalence of antibodies to Bovine Viral Diarrhea Virus (BVDV) in blood and milk serum in dairy cattle in Kars district of Turkey. *Indian Journal of Animal Research* **2016**, *50*, 811-815, doi:10.18805/ijar.9497.
300. Zanatto, D.C.S.; Gatto, I.R.H.; Labruna, M.B.; Jusi, M.M.G.; Samara, S.I.; Machado, R.Z.; Andre, M.R. Coxiella burnetii associated with BVDV (Bovine Viral Diarrhea Virus), BoHV (Bovine Herpesvirus), Leptospira spp., Neospora caninum, Toxoplasma gondii and Trypanosoma vivax in reproductive disorders in cattle. *Rev Bras Parasitol Vet* **2019**, *28*, 245-257, doi:10.1590/S1984-29612019032.
301. Zhang, J.; Wang, W.; Yang, M.; Lin, J.; Xue, F.; Zhu, Y.; Yin, X. Development of a One-Step Multiplex Real-Time PCR Assay for the Detection of Viral Pathogens Associated With the Bovine Respiratory Disease Complex. *Front Vet Sci* **2022**, *9*, 825257, doi:10.3389/fvets.2022.825257.

302. Zhang, K.; Zhang, J.; Qiu, Z.; Zhang, K.; Liang, F.; Zhou, Q.; Wang, L.; Li, J. Prevalence characteristic of BVDV in some large scale dairy farms in Western China. *Front Vet Sci* **2022**, *9*, 961337, doi:10.3389/fvets.2022.961337.
303. Zhong, F.; Li, N.; Huang, X.; Guo, Y.; Chen, H.; Wang, X.; Shi, C.; Zhang, X. Genetic typing and epidemiologic observation of bovine viral diarrhea virus in Western China. *Virus Genes* **2011**, *42*, 204-207, doi:10.1007/s11262-010-0558-4.
304. Zhou, Y.; Ren, Y.; Dai, G.; Li, X.; Xiang, Y.; Zhang, J.; Jiang, Y.; Jiang, S.; Hou, X.; Zhu, Z.; et al. Genetic characterization and clinical characteristics of bovine viral diarrhea viruses in cattle herds of Heilongjiang province, China. *Iran J Vet Res* **2022**, *23*, 69-73, doi:10.22099/IJVR.2021.38650.5625.
305. Zhou, Y.; Shao, Z.; Dai, G.; Li, X.; Xiang, Y.; Jiang, S.; Zhang, Z.; Ren, Y.; Zhu, Z.; Fan, C.; et al. Pathogenic infection characteristics and risk factors for bovine respiratory disease complex based on the detection of lung pathogens in dead cattle in Northeast China. *J Dairy Sci* **2023**, *106*, 589-606, doi:10.3168/jds.2022-21929.
